# Gut Microbiota Signatures Associated with Metastatic Prostatic Cancer Treated with Androgen Receptor Pathway Inhibitor (ARPI) Therapy

**DOI:** 10.3390/ijms27198454

**Published:** 2026-09-22

**Authors:** Sonia Pérez-Castro, Manuel Carballo-Quinta, Nahir Rodríguez-Costas, Adrián Freire-Rodríguez, Carlos Davina-Nunez, Alejandro Bellas-Pereira, Pablo Taboada-Rodríguez, Miguel Pérez-Schoch, Jorge J. Cabrera-Alvargonzález, Carlos A. Muller-Arteaga, Juan F. Sánchez-García, Enrique Cespón-Outeda, M. Elena López-Díez

**Affiliations:** 1 Microbiology Department, Complexo Hospitalario Universitario de Vigo (CHUVI), 36312 Vigo, Spain; jorge.julio.cabrera.alvargonzalez@sergas.es; 2 Microbiology and Infectology Research Group, Galicia Sur Health Research Institute (IIS Galicia Sur), 36312 Vigo, Spain; nahir.rodriguez@iisgaliciasur.es (N.R.-C.); carlosdavinha@hotmail.es (C.D.-N.); 3 Urology Department, Complexo Hospitalario Universitario de Vigo (CHUVI), 36312 Vigo, Spain; manuel.carballo.quinta@sergas.es (M.C.-Q.); adrian.freire.rodriguez@sergas.es (A.F.-R.); alejandro.bellas.pereira@sergas.es (A.B.-P.); miguel.perez.schoch@sergas.es (M.P.-S.); carlos.andres.muller.arteaga@sergas.es (C.A.M.-A.); juan.francisco.sanchez.garcia@sergas.es (J.F.S.-G.); enrique.cespon.outeda@sergas.es (E.C.-O.); 4 Uropathology Research Group, Galicia Sur Health Research Institute (IIS Galicia Sur), 36312 Vigo, Spain; 5 Functional Biology and Health Sciences Department, Universidade de Vigo, 36310 Vigo, Spain; 6 Escuela Técnica Superior de Ingeniería (ETSE), Universidade de Santiago de Compostela, 15782 Santiago de Compostela, Spain; pablo.taboada@gmail.com

**Keywords:** microbiome, metastatic prostate cancer, ARPIs, progression, prediction, *SCFA*

## Abstract

Metastatic prostate cancer (MPCa) remains an aggressive disease despite the introduction of androgen receptor pathway inhibitors (ARPIs), and biomarkers predicting progression are still lacking. The aim of this study was to investigate gut microbiota (GM) composition and functional potential associated with advanced disease and progression under ARPI therapy. The GM of 15 patients with metastatic PCa receiving ARPIs, including 5 patients with disease progression, was compared with that of 39 patients with untreated (UT) PCa using shallow shotgun metagenomic sequencing. The metastatic PCa cohort receiving ADT plus ARPI therapy showed reduced microbial alpha diversity and significant taxonomic and functional differences compared with the untreated, clinically localized PCa reference cohort. In patients with advanced disease, microbial functions related to dietary carbohydrate utilization, fermentation, epithelial interaction, and microbial structural maintenance were predominantly underrepresented, whereas stress-responsive and redox-associated functions were overrepresented. These alterations may reflect reduced metabolic and ecological versatility alongside increased adaptation to host-associated stress conditions. Compared with the UT group, in patients with progression we found that *Bifidobacterium longum, Anaerobutyricum hallii, Ruminococcus* sp., and *Blautia obeum* (LinDA coefficients 5.7, 2.6, 2.6 and 2.4, respectively) were enriched, whereas *Sutterella wadsworthensis, Butyricimonas, Bilophila wadsworthia, Alistipes indistinctus*, and *Intestinimonas* were depleted (LinDA coefficients −8.4, −5.9, −5, −5 and −3, respectively). Together, these findings identify distinct taxonomic and functional features associated with metastatic PCa under systemic therapy and microbial features observed in patients experiencing progression during ARPI treatment, providing potential candidates for biomarker development and generating testable hypotheses regarding microbiome–host metabolic interactions in treatment resistance. The GM may therefore represent a potential source of biomarkers for advanced PCa and progression under ARPI therapy, although longitudinal and multi-omics studies are required to determine their predictive value and functional relevance.

## 1. Introduction

Prostate cancer (PCa) ranks second globally in incidence among men and has even been predicted to increase by 65% from 2022 to 2045 worldwide on the basis of GLOBOCAN estimates for 2022 [1,2]. The mortality rate for PCa ranks fifth among men worldwide despite current treatment options [2], making PCa a great challenge for the healthcare system.

The combination of androgen deprivation therapy (ADT) and androgen receptor pathway inhibitors (ARPIs), which is associated with chemotherapy in selected cases, is the standard medical treatment in recurrent or metastatic disease [3]. During therapy, most patients may eventually develop castration-resistance [4], which is associated with a poor prognosis. Biomarkers are needed for early identification of relapse.

Several gut bacteria, which are involved in androgen biosynthesis [5], as well as oncogenesis or inflammation pathways [6], could play a relevant role in PCa biology. A correlation was observed between reduced fecal microbiota alpha-diversity and increased tumor burden in patients and mouse models [7]. In the same study, fecal microbiota transplantation from patients with high-volume PCa stimulated tumor growth in mice. The gut microbiome can affect PCa progression and treatment through microbiota-derived metabolites such as bacterial lipopolysaccharide (LPS), secondary metabolites, polyamines, short-chain fatty acids and microbial tryptophan catabolites [8].

Conversely, microbiota-targeted interventions could reduce PCa growth or modify treatment response. Oral administration of abiraterone, a key ARPI, has been shown to increase the relative abundance of *Akkermansia muciniphila*, a health-associated commensal, within the gut microbiota compared to other conventional treatments. This was associated with an increase in bacterial biosynthesis of vitamin K2, an inhibitor of androgen dependent and independent tumor growth [9]. First-generation non-steroidal anti-androgens such as bicalutamide, as well as ARPIs including enzalutamide and abiraterone acetate, may be associated with the increased abundance of taxa previously associated with response to anti-PD-1 immunotherapy, such as *Akkermansia muciniphila* and *Ruminococcus* spp. [10]. A recent study also reported that a dietary intervention with long-chain omega-3 fatty acids reduced PCa growth in mice and cancer up-grading in pre-prostatectomy PCa patients [7]. Specifically, this intervention was correlated with a reduction in gut *Ruminococcaceae* and fecal butyrate levels in PCa patients [7].

In this study, we hypothesized that gut dysbiosis may have an inflammatory potential and predict disease progression in patients with metastatic PCa undergoing ARPI therapy. This cohort included patients with metastatic hormone-sensitive and metastatic castration-resistant disease, all of whom were receiving ARPI-based therapy. To test this hypothesis, shallow shotgun sequencing was used to characterize the composition and functional potential of the gut microbiota.

## 2. Results

### 2.1. Clinical Characteristics of the Study Population

This study focuses on 15 patients with advanced disease (AD) who receive ARPI therapy and 39 patients with UT PCa. At the time of sampling, 10 of these 15 patients met the criteria for stable disease, while 5 met the criteria aligned with PCWG3 for biochemical and/or radiographic progression [11]. This group was histopathologically classified as Gleason 6/ISUP 1 (13.3%), Gleason 7/ISUP 2–3 (26.7%), or Gleason 8–10/ISUP 4–5 (60%). All underwent radical prostatectomy as primary treatment and 11 received salvage or adjuvant radiotherapy. The mean age was 76.3 years (standard deviation 10.9) and the mean BMI was 29.1. The demographic characteristics and dietary habits of the AD and UT groups were similar except for age and the frequency of beer or wine consumption (Table 1, Supplementary Table S1).

### 2.2. Metagenomic Sequencing Quality and Filtering

Shotgun metagenomic sequencing yielded a median depth of 18.2 million reads per sample (range: 9.7–46.1 million). Following quality filtration, the median read length was 133.1 bp (range: 82.4–142.8 bp). The high quality of the processed data was reflected in a median mapping of 91.1%. The negligible proportion of host DNA was confirmed by a low median abundance of sequences assigned to the Eukaryota Superkingdom (0.0031%). Taxonomic features were filtered to include only species with a minimum prevalence of 20%, resulting in 458 species retained for downstream analysis out of the 3492 initially detected.

### 2.3. Taxonomic Profile and Gut Microbiota Diversity in Advanced Disease

The metastatic PCa group receiving ADT plus ARPI therapy exhibited lower alpha diversity than the untreated, clinically localized PCa reference group, with significant reductions in the Observed and Chao1 indices (*p* = 0.003 and *p* = 0.001, respectively) (Figure 1b). Differences were robust to age adjustment (*p* = 0.01), while adjustment for radiotherapy (a factor closely linked to the AD group) resulted in a shared variance that limited the independent significance of AD (*p* > 0.05). Beta diversity differed significantly between the AD and UT groups based on Bray–Curtis and Jaccard distances (PERMANOVA, *p* = 0.001) (Figure 1c). These findings remained robust after adjusting for age (*p* = 0.002 and *p* = 0.004, respectively) or radiotherapy (*p* = 0.026), and maintained marginal significance when adjusting for both covariates simultaneously (*p* = 0.049).

### 2.4. Differential Taxonomic Abundance Associated with Advanced Disease and Progression

We assessed GM taxa differentially represented between the metastatic PCa cohort receiving ADT plus ARPI therapy and the untreated, clinically localized PCa reference cohort. A separate analysis was performed for patients experiencing progression under ARPI therapy, using the same untreated, clinically localized cohort as the reference group.

The GM taxa enriched in the AD group (N = 15) at the species level compared to the UT group are shown in Figure 2. Based on the LinDA linear model, *Alistipes merdavium*, *Mitsuokella*, *Lactobacillus*, *Intestinibacter* and *Clostridium perfringens* were enriched (coefficient 6.3, 5.2, 4.6, 3.4 and 3.1, respectively), while *Sutterella wadsworthensis*, *Butyricimonas*, *Alistipes indistinctus*, *Intestinimonas*, *Bilophila wadsworthia*, *Enterocloster*, *Flavonifractor plautii* were depleted (coefficient −5.9, −4.6, −3.3, −3, −2.6, −2.2 and −2.2, respectively) in the AD group (Supplementary Table S2).

To identify taxa associated with the progression phenotype among patients receiving ARPI-based therapy, an additional exploratory analysis was performed comparing the 5 patients with progression under ADT plus ARPI therapy with the 39 untreated, clinically localized PCa patients. *Bifidobacterium longum*, *Anaerobutyricum hallii*, *Ruminococcus* sp., *Blautia obeum* and *Faecalibacterium* were enriched in the progression stage (linDA coefficient 5.7, 2.6, 2.6, 2.4 and 1.5 respectively) whereas *Sutterella wadsworthensis*, *Butyricimonas*, *Bilophila wadsworthia*, *Alistipes indistinctus* and *Intestinimonas* were depleted (linDA coefficient −8.4, −5.9, −5, −5 and −3, respectively) (Supplementary Table S3). The obtained adjusted *p*-values are shown in Supplementary Figure S1.

*Alistipes indistinctus* lost significance when adjusted for age. When adjusting for radiotherapy, only the depletion of *Bilophila wadsworthia* remained significant (Supplementary Tables S4 and S5). All taxa identified as significantly associated with the progression stage in the LinDA analysis exhibited high prevalence across the cohort, being detected in 91–100% of the samples. Their distribution and abundance patterns can be visualized as the square-root transformed relative abundances for each sample in Supplementary Figure S2.

### 2.5. Differential Functional Potential of the Gut Microbiota Associated with Advanced Disease

To investigate the functional implications of these taxonomic shifts, we analyzed the metabolic potential of the GM. Two main comparisons were conducted using the UT group as the reference: advanced disease vs. untreated (AD vs. UT) and progression vs. untreated (PR vs. UT). In the AD vs. UT comparison, DESeq2 identified 125 significant orthologs ($p_{Adj}$ < 0.05 and $Log_{2}FoldChange$ > |1|). LinDA identified 144 significant orthologs. A total of 36 orthologs were consistently detected by both methods (Supplementary Table S6). In the PR vs. UT comparison, DESeq2 identified 120 significant orthologs, while LinDA identified 13 orthologs. Three orthologs were consistently identified by both methods (Supplementary Table S7). The results of the differential ortholog analysis (LinDA) are shown in Figure 3.

Genes involved in carbohydrate metabolism were underrepresented in the AD group. In particular, raffinose synthase (ortholog K06617, $p_{Adj}$ = 1.5033 × 10^−5^, $Log_{2}FoldChange$ = −5.01, EC 2.4.1.82) and sucrose-phosphate synthase (ortholog K00696, $p_{Adj}$ = 2.5137 × 10^−2^, $Log_{2}FoldChange$ = −3.26, EC 2.4.1.14), both associated with carbohydrate utilization and fermentative metabolism, were significantly depleted in AD (Supplementary Figures S3 and S4). Furthermore, the TGD transporter (ortholog K20023, $p_{Adj}$ = 1.0552 × 10^−2^, $Log_{2}FoldChange$ = −2.72), involved in galactarate/aldarate metabolism, was also underrepresented in the AD group. Likewise, enzymes participating in the butanoate metabolism pathway, including hydroxymethylglutaryl-CoA lyase (ortholog K01640, $p_{Adj}$ = 1.6605 × 10^−2^, $Log_{2}FoldChange$ = −1.46, EC 4.1.3.4) and 2-hydroxy-4-carboxymuconate semialdehyde hemiacetal dehydrogenase (ortholog K10219, $p_{Adj}$ = 4.0099 × 10^−2^, $Log_{2}FoldChange$ = −2.65, EC 1.1.1.-), were also underrepresented in the AD group (Supplementary Figure S5).

Regarding bacterial environmental sensing and stress-response pathways, two-component regulatory system analysis showed a underrepresentation of the FusR and TtrR genes (orthologs K20264 and K13041, adjusted $p_{Adj}$ = 2.1138 × 10^−5^ and 2.1135 × 10^−2^, $Log_{2}FoldChange$ = −4.74 and −2.37, respectively), whereas the MprB sensor kinase (ortholog K07653, $p_{Adj}$ = 1.4368 × 10^−2^, $Log_{2}FoldChange$ = 1.98) was significantly overrepresented in the AD group.

Genes associated with biofilm formation and cell envelope biosynthesis were also reduced in the AD group. Specifically, the biofilm-associated PslA gene (ortholog K20997, $p_{Adj}$ = 1.6605 × 10^−2^, $Log_{2}FoldChange$ = −2.13) and two enzymes involved in nucleotide sugar biosynthesis, HldE (ortholog K21345, $p_{Adj}$ = 7.7256 × 10^−3^, $Log_{2}FoldChange$ = −3.11) and Prt (ortholog K12453, $p_{Adj}$ = 4.1746 × 10^−2^, $Log_{2}FoldChange$ = −3.39, EC 1.1.1.-), were significantly underrepresented in AD (Supplementary Figures S5 and S6).

Functional differences in lipid, redox and cell envelope metabolism were also observed. The gene encoding 1,3-propanediol dehydrogenase (ortholog K00086, $p_{Adj}$ = 4.7715 × 10^−2^, $Log_{2}FoldChange$ = 1.13) was overrepresented in the AD group, whereas hydroxymethylglutaryl-CoA lyase (ortholog K01640, $p_{Adj}$ = 1.6605 × 10^−2^, $Log_{2}FoldChange$ = −1.46, EC 4.1.3.4) was underrepresented (Supplementary Figure S5). Furthermore, N-acetylmuramate phosphatase (MupP) (ortholog K22292, $p_{Adj}$ = 4.1746 × 10^−2^, $Log_{2}FoldChange$ = −2.45), involved in peptidoglycan recycling, was significantly underrepresented in AD.

Orthologs related to amino acid metabolism were also depleted in AD. In the histidine metabolism pathway, urocanate reductase (ortholog K17363, $p_{Adj}$ = 4.0401 × 10^−2^, $Log_{2}FoldChange$ = −1.79, EC 1.3.99.33) and N-formylglutamate deformylase (ortholog K14665, $p_{Adj}$ = 4.9880 × 10^−4^, $Log_{2}FoldChange$ = −2.54, EC 3.5.1.68) showed significantly lower representation in the AD group (Figure 4).

Finally, additional orthologs involved in cellular regulation, membrane functions and epithelial interaction were significantly underrepresented in AD, including the outer membrane porin GBP (ortholog K03285, $p_{Adj}$ = 3.6962 × 10^−3^, $Log_{2}FoldChange$ = −4.44), aconitate decarboxylase (ACOD1) (ortholog K17724, $p_{Adj}$ = 2.4936 × 10^−4^, $Log_{2}FoldChange$ = −4.09), the multidrug efflux protein MFP (ortholog K18901, $p_{Adj}$ = 3.4261 × 10^−2^, $Log_{2}FoldChange$ = −2.23), the CBS-domain protein (ortholog K07182, $p_{Adj}$ = 4.1948 × 10^−2^, $Log_{2}FoldChange$ = −1.64), and the EPS8-associated ortholog K17277 ($p_{Adj}$ = 1.4979 × 10^−2^, $Log_{2}FoldChange$ = −1.41).

## 3. Discussion

### 3.1. Taxonomic Changes Associated with Advanced Disease Under ARPI Therapy

The use of ARPIs for the treatment of metastatic castration-resistant and hormone-sensitive PCa has improved overall survival. Nevertheless, the prediction of response remains challenging. A reduction in PSA blood levels in the first month of ARPI treatment could be correlated with overall survival, but an increase in PSA is not always associated with a poor outcome. The search for biomarkers is needed to guide personalized treatment. We observed some differences in gut microbiota composition between the metastatic PCa cohort receiving ADT plus ARPI therapy and the untreated, clinically localized PCa reference cohort. Given the overlap between groups in the PCoA and the study design, these differences cannot be attributed independently to metastatic disease or systemic treatment. Rather, they may reflect a combination of tumor burden, metastatic status, host metabolic changes, and exposure to systemic therapy. Although universal signatures of a healthy or unhealthy gut microbiota remain elusive, it is well established that tumors can orchestrate compositional shifts to their own benefit [12]. Prototypic tumor-associated dysbiosis is frequently characterized by the overrepresentation of *Flavonifractor plautii* and the *Enterocloster* genus alongside a concurrent depletion of *Eubacterium* and *Lactobacillus* spp. [12]. Interestingly, our findings reveal a contrasting pattern: compared to the untreated PCa cohort, the group with advanced disease receiving ADT plus ARPI therapy, which included both hormone-sensitive and castration-resistant patients, exhibited a lower abundance of *Enterocloster* and *F. plautii*, coupled with a higher abundance of *Lactobacillus* spp. Therefore, this compositional pattern may reflect the combined influence of advanced disease and systemic therapeutic exposure, although our study design does not allow these effects to be disentangled. Furthermore, *Lactobacillus* spp. may be associated with castration-resistant prostate cancer [13].

The observed enrichment of *Alistipes* in the metastatic PCa cohort receiving ADT plus ARPI therapy is noteworthy. Previous studies have demonstrated the enrichment of *Alistipes* in the GM of mice after fecal microbiome transplantation from castration-resistant patients. *Alistipes* could promote PCa progression via the IGF-1 axis [14]. Rather than being inherently beneficial or harmful, the dual ecological role of *Alistipes* is highly context-dependent and governed by its metabolic output. Its impact on gut inflammation is largely determined by the delicate balance between the production of succinic acid and protective short-chain fatty acids (SCFAs). When external perturbations disrupt this metabolic equilibrium, the functional phenotype of *Alistipes* can shift toward pathogenicity. Consequently, profiling the specific metabolic signatures of *Alistipes* represents a highly valuable avenue for future research.

Concurrently, the metabolic toxicity often associated with these treatments could potentially contribute to the overrepresentation of metabolic disorder-associated genera like *Intestinibacter*, alongside the reduced abundance of commensal butyrate producers. ADT is known to induce systemic metabolic alterations, which could potentially influence the gut microbial environment. However, given the inherent confounding between metastatic disease and systemic treatment in our cohort, we cannot determine whether the observed microbiota differences are related to ADT, ARPI exposure, metastatic disease, or their combined effects.

### 3.2. Microbial Taxa Potentially Associated with Disease Progression Under ARPI Therapy

Among patients with metastatic PCa receiving ADT plus ARPI therapy, we observed differences in microbial taxa between patients with and without disease progression. However, because only five patients experienced progression and the cohorts also differed in disease status and treatment exposure, these findings should be considered exploratory and hypothesis-generating rather than specific signatures of progression or treatment resistance. Unlike other solid tumors, prostate cancer is heavily dependent on *de novo* lipogenesis. Elevated systemic acetate, which may be produced by the GM, could potentially be utilized by prostate cancer cells via the ACSS2 enzyme to support lipid synthesis and cell survival [15]. Furthermore, GM-derived acetate and other short-chain fatty acids have been shown to systemically promote PCa growth by driving IGF-1 signaling cascades [16]. *Bifidobacterium longum* [17] and *Blautia* spp. have been described as acetate producers through specific metabolic mechanisms [18]. The potentially context-dependent role of the genus *Bifidobacterium* has been previously discussed [19,20]. It is possible that different species within this genus may have distinct, and potentially opposing, associations with cancer biology. Indeed, specifically *Bifidobacterium dentium* has been reported to be enriched in patients with cancer [12]. Therefore, the increased abundance of *Bifidobacterium* observed among patients with progression should not necessarily be interpreted as a beneficial or protective feature, particularly given the limited number of progression cases and the species-specific differences within this genus.

The acetate cross-feeding hypothesis could also provide a potential explanation for the enrichment of some butyrate producers that can utilize acetate, such as *Anaerobutyricum hallii* or *Faecalibacterium* [21,22] among the patients with progression. However, given the small number of progression cases in our cohort (N = 5), these observations should be considered hypothesis-generating rather than evidence of a causal metabolic mechanism underlying disease progression.

Among patients receiving ADT plus ARPI therapy, we also observed the persistence or enrichment of *Ruminococcus*. Previous work has reported enrichment of *Ruminococcus* spp. in patients with castration-resistant PCa and proposed that their ability to synthesize dehydroepiandrosterone from pregnenolone may contribute to tumor growth in an androgen-deprived environment [5]. Although this provides a biologically plausible context for our observation, our data do not establish a mechanistic role for *Ruminococcus* in disease progression and further functional studies are required to determine whether this association reflects an adaptive microbial response, a consequence of the treatment environment, or a contribution to tumor progression.

Overall, our findings identify microbial taxa associated with advanced PCa in patients receiving ADT plus ARPI therapy, while the findings related specifically to disease progression should be interpreted cautiously because they are based on a limited number of progression cases. Rather than supporting specific microbial biomarkers of progression, these observations provide candidate taxa for further investigation. Future studies using larger and independent cohorts, together with specialized cohort comparisons, will be necessary to disentangle the potential contributions of age, radiotherapy, and first-line systemic treatments. Importantly, all patients in the metastatic cohort had previously undergone radical prostatectomy for clinically localized disease, suggesting that our cohort represents patients who subsequently developed metastatic disease following local treatment rather than patients with *de novo* metastatic PCa. This distinction may limit the generalizability of our findings and should be specifically addressed in future studies. Prospective longitudinal studies following patients from diagnosis before systemic treatment through ADT and ARPI exposure would be particularly informative. Comparisons between patients with *de novo* metastatic PCa before treatment and patients with metastatic disease emerging after prior local therapy could further help distinguish microbiome alterations associated with metastatic disease from those related to systemic treatment. Functional and longitudinal studies, ideally integrating metabolomic measurements, will also be important to determine whether the observed microbial signatures are associated with altered acetate and other metabolite levels and whether they contribute to disease progression.

### 3.3. Functional Changes in the Gut Microbiota in Advanced Disease

Rather than implying deterministic microbiome-driven effects on disease progression, these findings should be interpreted as a shift in the functional potential of microbial communities associated with distinct host metabolic and therapeutic contexts. The cross-sectional design precludes causal inference; therefore, observed differences likely reflect ecological adaptation to tumor burden, host metabolic remodeling, and treatment-associated selective pressures.

A first major axis of differentiation in GM was observed in carbohydrate metabolism and dietary polysaccharide utilization, which was predominantly underrepresented in the metastatic, treated group. Underrepresented enzymes such as raffinose synthase (K06617) and sucrose-phosphate metabolism-related functions (K00696) suggest a microbiome with decreased capacity for complex carbohydrate processing. These pathways are generally associated with fermentation processes leading to short-chain fatty acid (SCFA) production, which has been widely linked to intestinal epithelial homeostasis and anti-inflammatory effects in the gut environment [23,24,25]. Their underrepresentation in AD samples may therefore indicate a reduced potential for SCFA production and, consequently, a diminished contribution of these microbial functions to intestinal homeostasis. Additional underrepresentation of galactarate/aldarate metabolism (K20023) and aromatic compound degradation (K10219) may further suggest reduced microbial capacity for the utilization of dietary substrates [26,27,28]. The reduced representation of microbial carbohydrate-processing functions may also have implications for the production of microbial exopolysaccharides (EPSs), which can contribute to intestinal barrier function and modulation of host immune responses [29]. Overall, this pattern may reflect a microbiome with reduced capacity for dietary carbohydrate utilization and fermentative metabolism, potentially limiting SCFA- and EPS-associated contributions to epithelial homeostasis and host–microbiome communication.

In contrast, a differential pattern was observed within microbial environmental sensing and regulatory systems. FusR (K20264) and TtrR (K13041) were underrepresented in AD samples, whereas MprB (K07653) was overrepresented in AD. FusR, linked to fucose sensing and utilization, has been associated with bacterial colonization dynamics in mucosal environments [30]. Its underrepresentation in AD may indicate reduced capacity to sense and respond to host-derived glycans, potentially altering microbial adaptation to the mucosal environment. Similarly, the underrepresentation of TtrR, involved in tetrathionate respiration, may reflect reduced metabolic versatility under fluctuating redox conditions [31,32,33]. Together, the reduced representation of FusR and TtrR may indicate a diminished repertoire of environmental sensing and stress-adaptation mechanisms in AD-associated microbiomes.

On the other hand, MprB (K07653), a sensor histidine kinase of the OmpR family, has been implicated in bacterial stress responses, including antimicrobial peptide resistance, virulence regulation, and adaptation to inflammatory environments [34,35,36]. Its overrepresentation in AD samples may indicate increased representation of stress-responsive regulatory networks in the context of an altered host-associated environment. Because MprB-related signaling has been linked to bacterial adaptation to host stress conditions, its increased representation may be consistent with greater microbial pressure to adapt to inflammatory or otherwise unfavorable ecological conditions. In parallel, alterations in microbial histidine metabolism may be relevant to the inflammatory context, as histidine can be converted to histamine by microbial histidine decarboxylase activity. Histamine is a biologically active mediator with immunomodulatory and, in specific tumor contexts, potentially pro-tumorigenic effects [37,38]. Thus, the combined alteration of stress-responsive regulation and histidine-associated functions may warrant consideration as a potential contributor to a more inflammatory microbial environment in AD, although direct changes in histamine production cannot be inferred from metagenomic representation alone.

Biofilm-associated and persistence-related functions also showed a consistent underrepresentation in AD samples. Notably, PslA (K20997), involved in exopolysaccharide biosynthesis and biofilm formation, was significantly underrepresented in AD. Importantly, recent evidence suggests that microbial polysaccharide-associated pathways may correlate with improved immunotherapy response and more effective tumor control [39,40]. Therefore, reduced PslA representation may indicate a decrease in microbial functions associated with biofilm-associated structural organization and polysaccharide-mediated host interaction. This could be relevant to the ecological stability and immunological properties of the gut microbiome, although the specific contribution of PslA to these processes in prostate cancer remains to be established.

A second relevant axis involved microbial lipid and redox metabolism. AD samples showed overrepresentation of 1,3-propanediol dehydrogenase (K00086), an enzyme involved in microbial redox homeostasis supporting bacterial survival under stressful anaerobic conditions [41,42,43]. Its overrepresentation in AD may reflect selection for microbial functions supporting redox flexibility and competitive advantage under more restrictive or inflammatory ecological conditions.

Conversely, hydroxymethylglutaryl-CoA lyase (K01640), involved in ketone body and amino acid degradation pathways, was underrepresented in AD samples, potentially indicating a reduced contribution of amino acid-derived carbon metabolism. This enzyme generates acetyl-CoA and acetoacetate, intermediates that can feed central metabolic networks and may indirectly contribute to SCFA pools through microbial cross-feeding interactions [44,45]. Thus, its reduced representation may reflect lower potential for fermentative energy production and nutrient-derived metabolic integration in AD microbiomes. More specifically, reduced microbial HMGCL representation could theoretically decrease the capacity for microbial ketogenesis while limiting leucine catabolism. However, this interpretation remains mechanistic and hypothetical, as no direct experimental evidence currently demonstrates that depletion of microbial HMGCL alone causes systemic leucine accumulation or reduced ketone-body availability in prostate cancer. Nevertheless, given the established links between leucine–mTORC1 signaling, ketone-body biology, and tumor progression, this functional alteration may represent a metabolically relevant feature of the AD-associated microbiome and a potential contributor to treatment resistance.

In parallel, enzymes involved in peptidoglycan recycling and nucleotide sugar biosynthesis, such as N-acetylmuramate phosphatase (K22292) and D-glycero-heptose adenylyltransferase (K21345), were underrepresented in AD samples. These functions are involved in bacterial cell wall recycling and biosynthesis, and their reduced representation may suggest decreased capacity for structural remodeling and maintenance of bacterial cell wall integrity, potentially contributing to a less stable microbial ecosystem and altered host–microbiome immune interactions [46,47].

Finally, a consistent underrepresentation of genes involved in immunometabolic and epithelial-interaction-related functions was observed in AD. Aconitate decarboxylase (K17724), linked to itaconate metabolism, is involved in pathways integrating microbial metabolic activity with host immune regulation and microbial–host immunometabolic interactions [48]. Its reduced representation in AD may suggest altered microbial contributions to these immunometabolic processes. Similarly, urocanate reductase (K17363) has been associated with modulation of immune-suppressive myeloid cell recruitment in tumor-associated inflammatory environments [49]. Its underrepresentation in AD may therefore reflect altered microbial potential to modulate host immune responses within the tumor-associated inflammatory environment. More broadly, altered representation of histidine-associated functions may affect the balance between microbial histidine catabolism and alternative pathways such as histamine production. Given the potential pro-inflammatory and immunomodulatory effects of histamine, this represents a plausible mechanistic link between altered microbial metabolism and the inflammatory context of AD, but would require metabolomic validation to establish.

Importantly, EPS8-associated proteins (K17277), outer membrane porins (K03285), CBS-domain proteins (K07182), and multidrug efflux systems (K18901) were underrepresented in AD samples, potentially indicating reduced microbial capacity for epithelial interaction, metabolic regulation, and environmental adaptability [50,51,52,53,54]. Rather than assigning a specific functional consequence to each of these heterogeneous protein families, their collective underrepresentation may be interpreted as further evidence of a broader reduction in the functional repertoire available for microbial environmental sensing, nutrient exchange, and host–microbiome interaction in AD.

Functional analysis of the five progressing patients revealed only three significantly altered genes. Because these genes do not map to the same metabolic pathway, they provide little functional insight, precluding any meaningful biological conclusions. This lack of convergence further emphasizes the need for caution when interpreting individual functional markers and suggests that the most informative signal in the present dataset lies in the broader functional patterns observed across the cohort rather than in isolated genes.

Taken together, these results suggest that AD-associated microbiomes exhibit reduced representation of functions related to metabolic flexibility, dietary substrate utilization, fermentative metabolism, epithelial support, and regulated host–microbiome interactions, alongside an increased representation of stress-responsive and redox-associated regulatory functions. This overall shift may reflect reduced functional versatility and a greater representation of microbial traits compatible with environmental and host-associated stress, rather than a discrete disease-specific microbial phenotype.

### 3.4. Study Limitations and Future Directions

Metagenomic functional inference reflects genetic potential rather than actual enzymatic activity. Therefore, conclusions regarding metabolic activity or host impact remain hypothetical without complementary metabolomic or transcriptomic validation. In particular, proposed links involving SCFA availability, histamine production, ketone-body metabolism, leucine accumulation, and treatment resistance should be considered testable mechanistic hypotheses rather than demonstrated consequences of the observed metagenomic differences.

Future studies integrating multi-omics approaches and longitudinal sampling will be essential to determine whether these functional signatures represent biomarkers of disease progression, consequences of androgen deprivation therapy, or adaptive microbial responses to tumor-associated metabolic reprogramming. Integration with targeted metabolomics would be particularly valuable for determining whether the observed functional differences translate into altered levels of SCFAs, histidine-derived metabolites, ketone bodies, and branched-chain amino acids, while longitudinal sampling could help distinguish treatment-associated ecological shifts from microbiome features linked to disease progression.

The main limitations of this study include the small cohort size and, importantly, the inherent confounding between disease status and treatment exposure in the study design. All patients in the metastatic cohort were receiving ADT in combination with an ARPI, whereas the untreated reference group consisted exclusively of patients with clinically localized PCa, including individuals under active surveillance or scheduled for prostatectomy. Moreover, the groups differed substantially in clinical characteristics, including ISUP grade and prior radiotherapy exposure, which are interrelated with disease stage and treatment status. Accordingly, the observed reduction in alpha diversity and the differences in gut microbiota composition and functional potential should be interpreted within this complex clinical phenotype and cannot be attributed independently to metastatic disease, tumor burden, or ADT/ARPI exposure. Rather than demonstrating a single microbiome-driven mechanism of ARPI resistance, the observed convergence of alterations in carbohydrate metabolism, SCFA-related functions, microbial redox metabolism, HMGCL-associated pathways, and host-interaction functions provides a coherent hypothesis of altered host–microbiome metabolic crosstalk in metastatic disease under systemic treatment. These findings should therefore be considered hypothesis-generating rather than disease-specific or treatment-specific signatures. Larger, prospectively designed and clinically stratified cohorts, including appropriate comparisons of untreated metastatic patients and treated localized patients, together with longitudinal sampling before and after initiation of ADT/ARPI therapy, will be necessary to disentangle the effects of disease progression from those of systemic treatment. Functional validation at the metabolite and transcriptional levels will also be required to determine whether the observed metagenomic differences translate into biologically relevant changes in microbial activity and host–microbiome interactions.

## 4. Materials and Methods

### 4.1. Clinical Cohort and Study Design

The study protocol (100/2022) was approved by the Galician network of committees of research ethics. Age, Gleason grade stratified into three subgroups (ISUP 1, 2–3, and 4–5), PSA levels at the time of sampling, presence of metastasis, radiotherapy treatment, body mass index (BMI), lifestyle habits (tobacco, alcohol, diabetes and hypercholesterolaemia treatment), follow-up time, and diet habits were collected. The patients did not receive antibiotic treatment in the previous 3 months.

A total of 54 patients were included: 15 with metastatic disease (advanced disease, AD) receiving a combination of ADT and ARPI and 39 with UT PCa (reference group). The AD group included patients with radiologically confirmed metastatic prostate cancer under continuous ADT in combination with ARPIs. Two AD subgroups were considered: 10 patients with stable disease (hormone-sensitive disease) and 5 patients with disease progression (castration-resistant disease). In this cohort, all patients classified as having stable disease were hormone-sensitive, whereas all patients classified as having progression were castration-resistant. Stable disease corresponded to the absence of clinical, biochemical, or radiological progression during ARPI therapy, including: no PSA increase ≥25% from nadir with an absolute rise ≥2 ng/mL (confirmed after ≥3 weeks), no radiographic progression on conventional imaging (CT and/or bone scan), and no clinical deterioration attributable to disease. Progression was established according to Prostate Cancer Working Group 3 (PCWG3) criteria, based on biochemical and/or radiographic evidence. PSA progression required a ≥25% increase from nadir (minimum absolute increase of 2 ng/mL), confirmed after ≥3 weeks. Radiographic progression was defined as the appearance of ≥2 new bone lesions or progression of soft tissue disease according to RECIST 1.1 criteria. Fecal samples from the 5 patients classified in the progression group were collected at the time when progression was clinically or radiographically confirmed and prior to any modification of systemic therapy. The UT patients were the reference group, including 29 patients on active surveillance and 10 patients scheduled for prostatectomy; samples were collected prior to surgery.

The study design inherently couples disease status and treatment exposure: all patients in the AD group had radiologically confirmed metastatic disease and were receiving continuous ADT in combination with an ARPI, whereas all UT patients had clinically localized PCa and were not receiving systemic treatment. Therefore, the comparison between AD and UT groups should be interpreted as a comparison between metastatic, systemically treated PCa and untreated, clinically localized PCa, rather than as an isolated assessment of the effect of disease stage or treatment. The UT group comprised patients managed with active surveillance or scheduled for prostatectomy, and consequently represents a clinically distinct population from the metastatic cohort.

### 4.2. Sample Collection and Sequencing

Fecal samples were collected in a transport medium (MGIEasy Stool Sample Collection kit, MGI Tech, Shenzhen, China). They were stored frozen at −80 °C within the first 24 h. Shallow shotgun sequencing was performed with Illumina DNA Prep (Illumina Inc., San Diego, CA, USA) in an Illumina NextSeq 1000 sequencer (Illumina Inc., San Diego, CA, USA).

### 4.3. Metagenomic Data Processing and Analysis

Metagenomic sequences were processed using the SqueezeMeta v1.6.3 pipeline (https://github.com/jtamames/SqueezeMeta, accessed on 9 August 2026). Quality control and adapter trimming were performed using Trimmomatic v0.39 with default parameters. Co-assembly of the filtered reads was executed using Megahit v1.2.9, and short contigs (<200 bp) were removed via prinseq v0.20.4. Following standard practices for fecal metagenomics, specific host read removal was not performed, as human DNA contamination in such samples is typically negligible. Taxonomic and functional assignments were conducted through a multi-step approach. ORFs were predicted using Prodigal v2.6.3, and similarity searches were performed against the GenBank (NCBI nr), eggNOG, and KEGG databases using Diamond v2.2.1. Additionally, 16S rRNA sequences were classified with the RDP classifier v2.14, and HMM homology searches against the Pfam database were performed using HMMER3 v3.4.

### 4.4. Taxonomic and Functional Annotation and Statistical Analysis

Student’s *T*-test was used to analyze continuous data from two clinical groups. ANOVA or Kruskal-Wallis, as appropriate, were used for comparison among three or more groups. The chi-square test was used to compare categorical data. The Pearson or Spearman test was used for correlation analysis, as appropriate. A *p* < 0.05 was statistically significant.

Low prevalent (<20%) and low abundant (<100 reads) species were removed. Metabolic pathway predictions for the KEGG and MetaCyc databases were executed using MinPath v1.6. For functional comparisons, gene abundances were normalized to copy numbers (the TPM of the gene divided by the TPM of universal single-copy genes) using the exportPathway function in SQMtools v1.8.0. $Log_{2}FoldChange$ were calculated based on these normalized values for KEGG map visualizations. The MicrobiomeStat R package (v1.4, https://github.com/cafferychen777/MicrobiomeStat, accessed on 9 August 2026) was used for the following comparisons between groups: Alpha diversity was assessed via linear models and beta diversity was compared using PERMANOVA using the vegan R package (v2.7-6, visualized via PCoA), both adjusting for potential confounders. Feature-level differential abundance was identified using the LinDA methodology [55] with Bonferroni-adjusted *p*-values, accounting for relevant confounding factors for sensitivity analysis.

To achieve a more granular functional characterization of the gut microbiome, the initial pathway-level KEGG enrichment analysis was complemented and refined by a differential abundance analysis at the level of KEGG Orthology (KO) identifiers. Ortholog profiles were extracted from the SQM object and filtered using a minimum prevalence threshold of 5% across samples. Differential abundance analysis was performed using two complementary statistical approaches: DESeq2 v1.50.2 and LinDA (Linear Models for Differential Abundance Analysis) function from MicrobiomeStat v1.4, applied to raw count data (Abund). In both approaches, statistical significance was defined as an adjusted *p*-value (FDR) < 0.05 and an absolute $Log_{2}FoldChange$ > 1.

To increase robustness and minimize methodological bias, only orthologs identified as significantly different by both DESeq2 and LinDA were selected for downstream functional interpretation. The directionality of differential abundance was defined according to the sign of the $Log_{2}FoldChange$, with positive values indicating overrepresentation in AD or PR groups and negative values indicating overrepresentation in the UT group. The functional annotation of selected orthologs was performed using the KEGG database, including pathway mapping and metabolic classification, and further interpreted through manual curation based on the current literature to contextualize their role in microbial metabolism, host–microbiome interactions, and cancer-related biological processes.

For the graphical representation of the identified orthologs, the symbol names associated with each KO in the KEGG database were retained as the official KEGG-associated nomenclature. These symbols were used solely as labels to facilitate the visualization of the corresponding KOs and do not represent the units used for differential analysis, which were the KO identifiers. For biological interpretation, orthologs with uncertain, poorly characterized, or ambiguous functional annotations, as well as those without a direct correspondence to a bacterial protein or function, were excluded from the functional interpretation. Only KOs with a clear and biologically relevant bacterial functional annotation were considered for downstream biological interpretation.

## 5. Conclusions

Our findings reveal differences in the taxonomic and functional landscape of the gut microbiota between patients with metastatic PCa receiving ADT plus ARPI therapy and an untreated, clinically localized PCa reference cohort. These differences encompass reduced representation of functions related to dietary substrate utilization, fermentation, microbial structural maintenance, and host–microbiome interactions, alongside increased representation of stress-responsive and redox-associated functions. Together with the distinct microbial taxa associated with disease progression under ARPI therapy, these findings highlight the potential of the gut microbiota as a source of candidate biomarkers and generate testable hypotheses regarding microbiome–host metabolic interactions in metastatic PCa under systemic treatment. However, given the inherent confounding between disease stage and treatment exposure in our cohort, the observed microbiome alterations cannot be attributed independently to metastatic disease, tumor burden, or ADT/ARPI exposure, and should therefore not be interpreted as disease-specific or treatment-specific signatures. The functional consequences and predictive relevance of these alterations require validation in larger, clinically matched and longitudinal cohorts integrating metagenomic, transcriptomic, and metabolomic approaches. Such studies will be essential to determine the independent contribution of disease progression and ADT/ARPI exposure and to establish the potential of these microbial signatures as biomarkers of disease progression and treatment response.

## Figures and Tables

**Figure 1:**
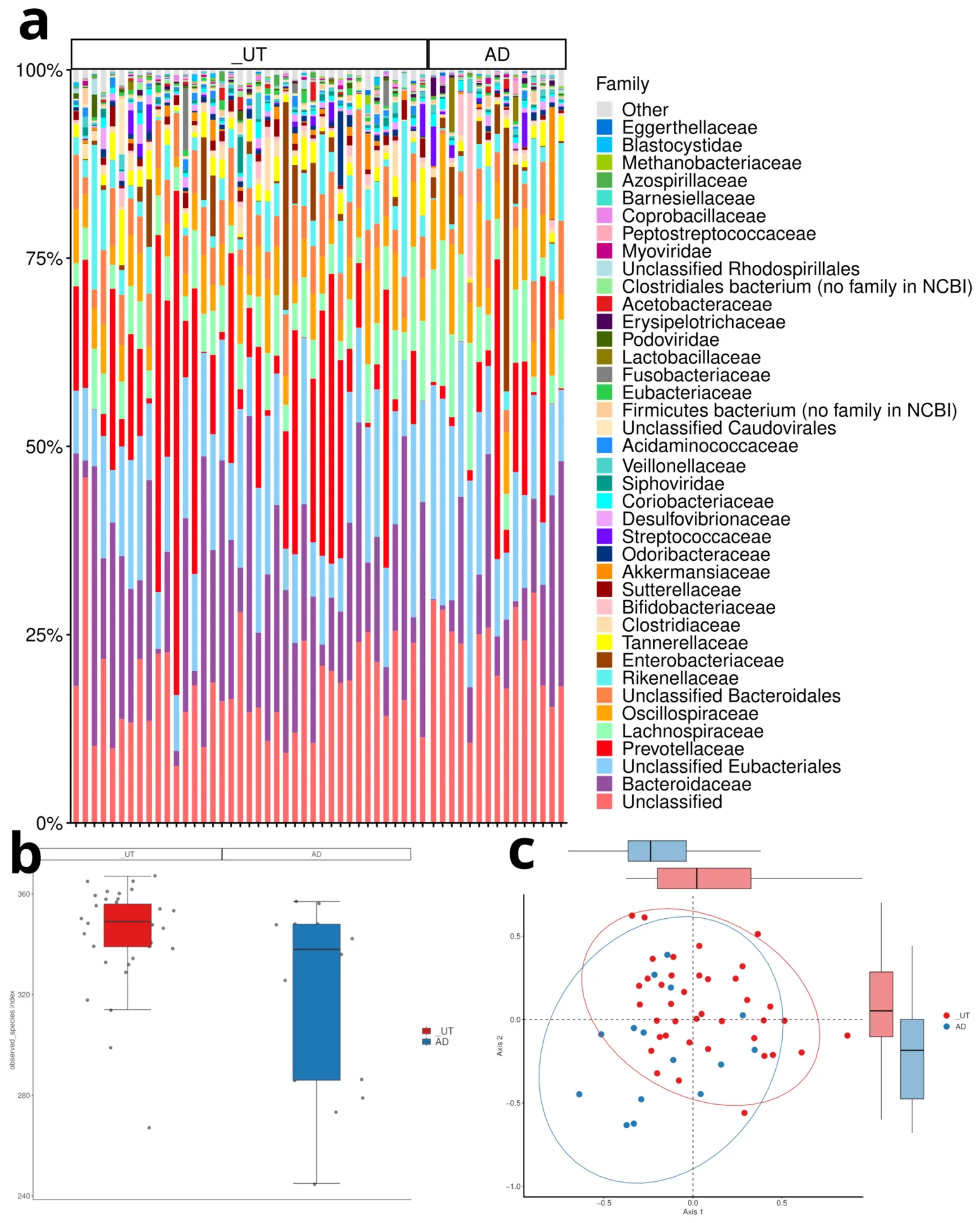
Gut microbiome characteristics in advanced disease (AD) vs. untreated patients (UT). (**a**) Taxa distribution at a family level. (**b**) Observed alpha diversity. (**c**) Principal Coordinate Analysis (PCoA) plot, based on Bray–Curtis beta diversity. Ellipses indicate the 95% confidence intervals for the control group (red) and the advanced disease group (blue).

**Figure 2:**
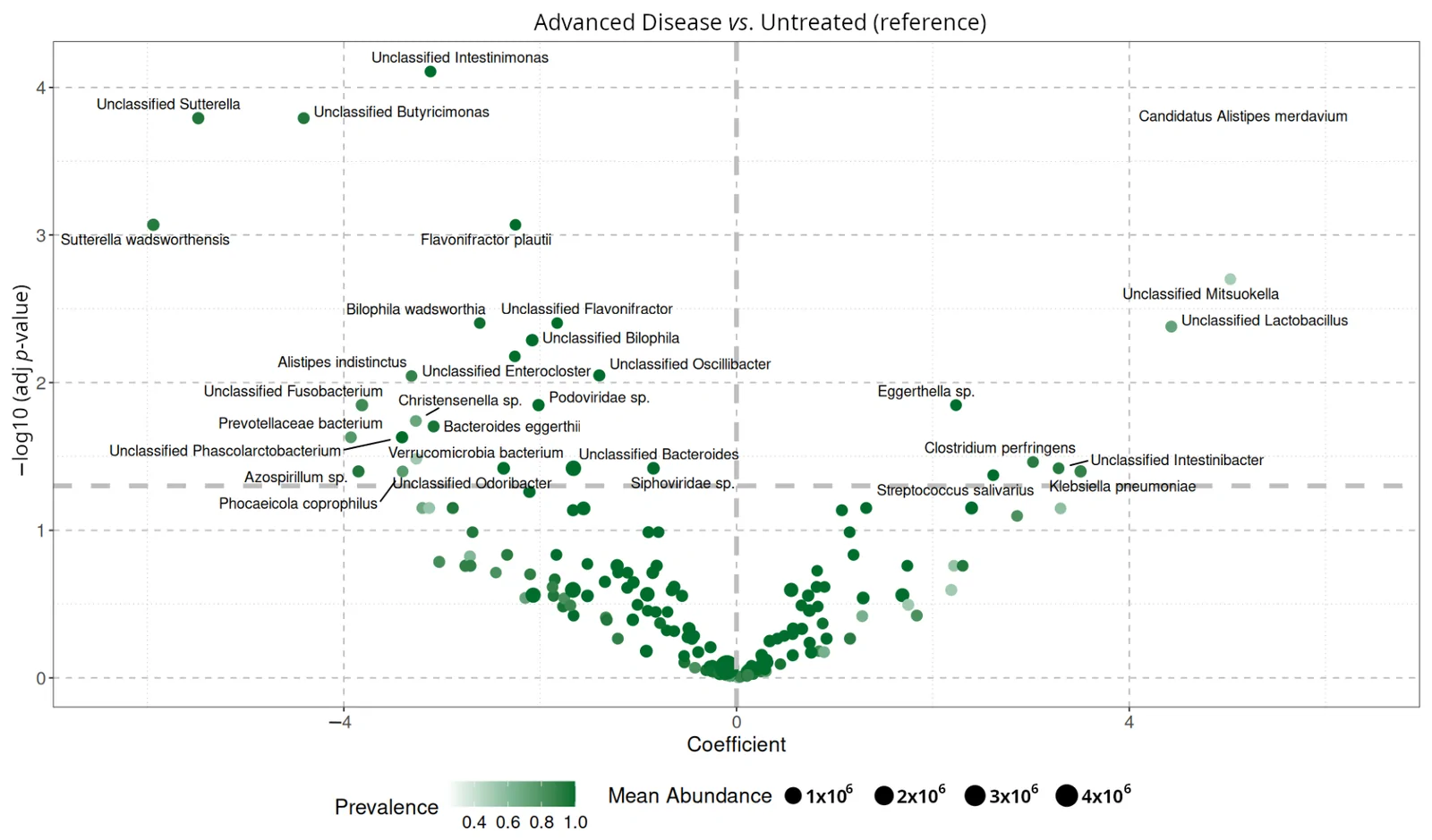
Gut microbiota taxa differentially represented between the metastatic PCa group receiving ADT plus ARPI therapy (N = 15) and the untreated, clinically localized PCa group (UT). Volcano plot of adjusted *p*-values obtained by Linear Discrimination Analysis (LinDA) on the y-axis. The x-axis shows the coefficient: Positive coefficients indicate higher abundance in the metastatic, treated group, whereas negative coefficients indicate higher abundance in the untreated, clinically localized group. The horizontal dashed line indicates the significance threshold of p<0.05 after Bonferroni correction ($-\log_{10}\left(0.05\right)\approx 1.30$).

**Figure 3:**
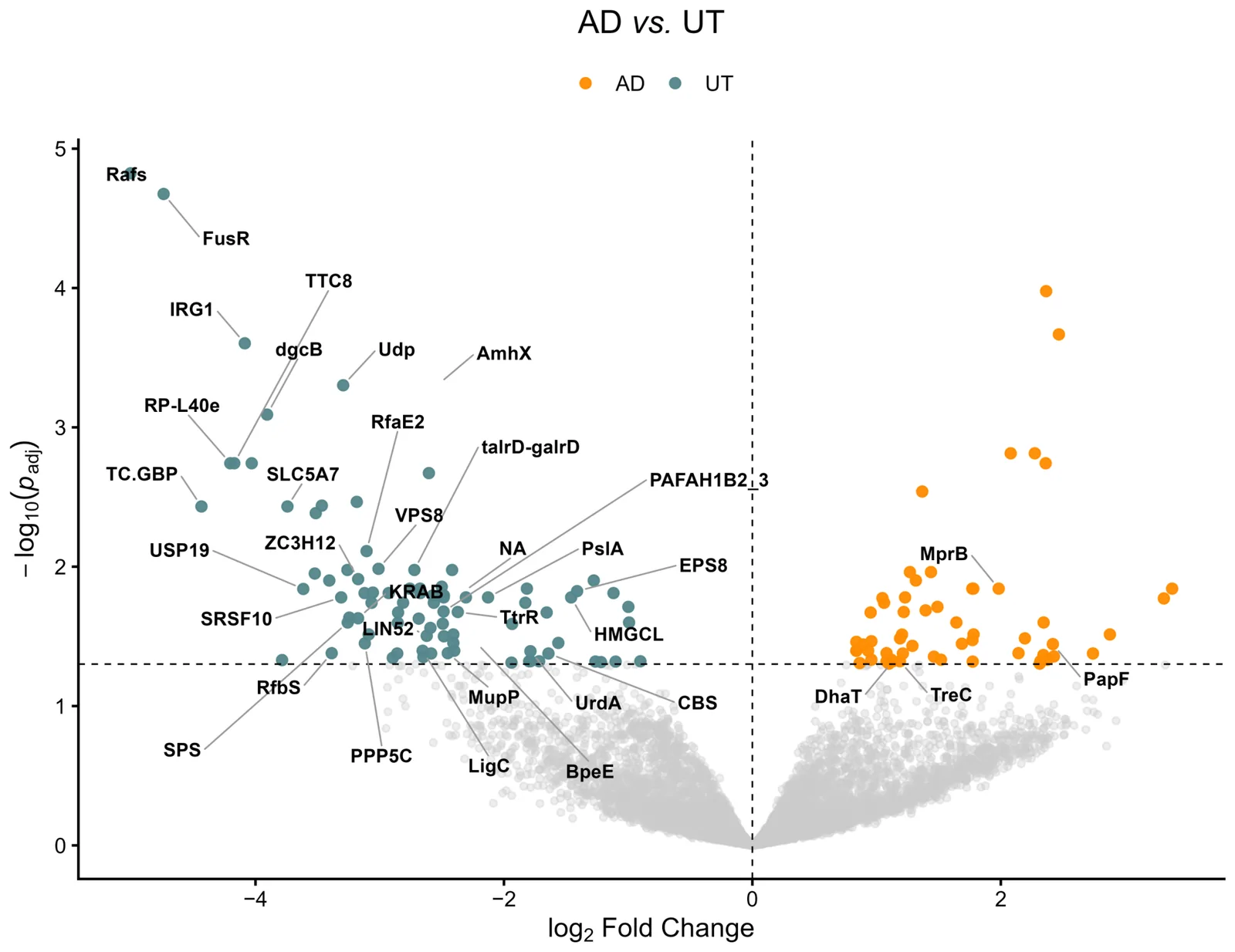
Differential abundance analysis of KEGG Orthologs (KOs) identified by LinDA. Orthologs exceeding the statistical significance threshold ($p_{Adj}$ < 0.05) are shown in blue or orange. Positive $Log_{2}FoldChange$ values (orange) correspond to overrepresented orthologs in AD, and negative values (blue) to underrepresented orthologs in AD. The symbols shown correspond to the official KEGG-associated symbols for the respective KO entries and they appears in 36 overlapping orthologs between LinDA and DESeq2. The correspondence between the symbol KEGG and the ortholog is shown in Supplementary Table S6.

**Figure 4:**
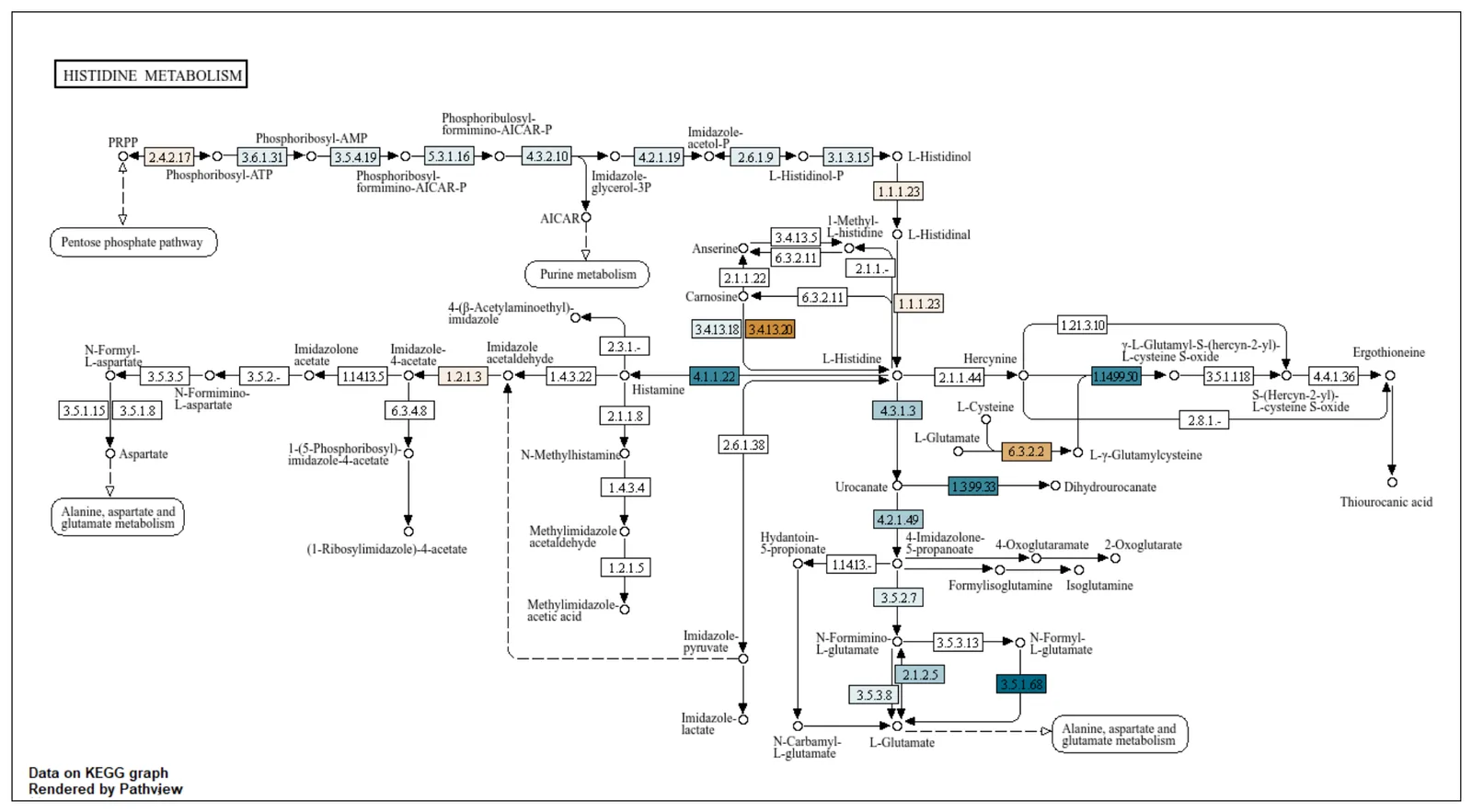
Histidine metabolism pathway (map00340). Positive $Log_{2}FoldChange$ values (orange) correspond to overrepresented orthologs in AD, and negative values (blue) to underrepresented orthologs in AD. Enzymes significantly underrepresented in AD patients (1.3.99.33 and 3.5.1.68) are shown in blue ($p_{Adj}$ = 4.0401 × 10^−2^ and $p_{Adj}$ = 4.9880 × 10^−4^, respectively).

**Table 1:** Characteristics of the studied population. Comparison of patients with advanced disease vs. untreated controls. PSA = Prostate Specific Antigen; ARPI = Androgen Receptor Pathway Inhibitor; SD: Standard Deviation.

	Advanced Disease	Untreated	*p*-Value
	N = 15	N = 39	
Age (mean (SD))	76.3 (10.9)	68.0 (7.49)	0.014
**Biopsy Gleason**			
Gleason 6/ISUP 1	2 (13.3%)	27 (69.2%)	
Gleason 7/ISUP 2–3	4 (26.7%)	10 (25.6%)	
Gleason 8–10/ISUP 4–5	9 (60%)	2 (5.13%)	
PSA at sampling (ng/mL)	10.9 (22.3)	4.85 (2.48)	0.312
**ARPI**			
Abiraterone	6 (40%)	0	
Enzalutamide	6 (40%)	0	
Apalutamide	3 (20%)	0	
ARPI time (months, mean (SD))	17.9 (15.5)		
**Radiotherapy**			<0.001
No	4 (26.7%)	39 (100%)	
Yes	11 (73.3%)	0	
BMI	29.1 (5.16)	28.2 (3.63)	0.553
**Hypertension**			0.787
No	6 (40%)	19 (48.7%)	
Yes	9 (60%)	20 (51.3%)	
**Tobacco**			0.818
Active Smoker	2 (13.3%)	3 (7.69%)	
Former Smoker < 10 years	2 (13.3%)	4 (10.3%)	
Former Smoker > 10 years	6 (40%)	16 (41%)	
Never Smoker	5 (33.3%)	16 (41%)	
**Diabetes treatment**			1
No	11 (73.3%)	27 (69.2%)	
Yes	4 (26.7%)	12 (30.8%)	
**Statins**			0.866
No	7 (46.7%)	21 (53.8%)	
Yes	8 (53.3%)	18 (46.2%)	

## Data Availability

The datasets generated and analyzed during the current study are available in the European Nucleotide Archive (ENA) repository (accession number PRJEB84128, ERR15104649-ERR15104705) (Supplementary Table S8). https://www.ebi.ac.uk/ena/browser/view/PRJEB84128 (accessed on 9 April 2026). All data generated in this study are included in the manuscript, Supplementary Tables S1–S8, and Supplementary Figures S1–S6. Any data not available in the paper or the repository is available from the corresponding author on reasonable request.

## Supplementary Materials

The following supporting information can be downloaded at: https://www.mdpi.com/article/10.3390/ijms27198454/s1.
