# Supplementary material for: Gut Microbiota Signatures Associated with Metastatic Prostatic Cancer Treated with Androgen Receptor Pathway Inhibitor (ARPI) Therapy
Source: Int J Mol Sci. 2026 Sep 22;27(19):8454. doi: 10.3390/ijms27198454 (PMC13665080; doi:10.3390/ijms27198454)
Supplement: Supplementary file 1 [file ijms-27-08454-s001.zip › ijms-4531381-supplementary.pdf]

Article

Gut Microbiota Signatures Associated with Advanced Metastatic Prostatic Cancer Treated with Androgen Receptor Pathway Inhibitor (ARPI) Therapy

Sonia Pérez-Castro [1,2,*], Manuel Carballo-Quinta [3,4], Nahir Rodríguez-Costas [2,5], Adrián Freire-Rodríguez [3], Carlos Davina-Nunez [2,5], Alejandro Bellas-Pereira [3], Pablo Taboada-Rodríguez [6], Miguel Pérez-Schoch [3], Jorge J. Cabrera-Alvargonzález [1,2], Carlos A. Muller-Arteaga [3,4], Juan F. Sánchez-García [3], Enrique Cespón-Outeda [3] and M. Elena López-Díez [3,4,*]

1 Microbiology Department, Complexo Hospitalario Universitario de Vigo (CHUVI), 36312 Vigo, Spain; jorge.julio.cabrera.alvargonzalez@sergas.es (J.J.C.-A.)
2 Microbiology and Infectology Research Group, Galicia Sur Health Research Institute (IIS Galicia Sur), 36312 Vigo, Spain; carlosdavinha@hotmail.es (C.D.-N.); nahir.rodriguez@iisgaliciasur.es (N.R.-C.)
3 Urology Department, Complexo Hospitalario Universitario de Vigo (CHUVI), 36312 Vigo, Spain; manuel.carballo.quinta@sergas.es (M.C.-Q.); adrian.freire.rodriguez@sergas.es (A.F.-R.); alejandro.bellas.pereira@sergas.es (A.B.-P.); miguel.perez.schoch@sergas.es (M.P.-S.); carlos.andres.muller.arteaga@sergas.es (C.A.M.-A.); juan.francisco.sanchez.garcia@sergas.es (J.F.S.-G.); enrique.cespon.outeda@sergas.es (E.C.-O.)
4 Uropathology Research Group, Galicia Sur Health Research Institute (IIS Galicia Sur), 36312 Vigo, Spain.
5 Functional Biology and Health Sciences Department, Universidade de Vigo, 36310 Vigo, Spain.
6 Escuela Técnica Superior de Ingeniería (ETSE), Universidade de Santiago de Compostela, 15782, Santiago de Compostela, Spain; pablo.taboada@gmail.com (P.T.-R.)
* Correspondence: sonia.maria.perez.castro@sergas.es (S.P.-C.); maria.elena.lopez.diez@sergas.es (M.E.L.-D.)

1. Supplementary material

Table S1. Characteristics of the studied population for dietary habits comparison between the untreated (UT, N = 39) and advanced disease (AD, N = 15) groups. The *p*-values for the chi-square test are shown.

	UT (N = 39)	AD (N = 15)	*p* overall
**Beer**			<0.001
> once/day	1 (2.56%)	0 (0.00%)	
1–2 times/week	16 (41.0%)	0 (0.00%)	
1–3 times/week	8 (20.5%)	4 (33.3%)	
3 or more times/week	6 (15.4%)	0 (0.00%)	
Daily	6 (15.4%)	0 (0.00%)	
Never	2 (5.13%)	8 (66.7%)	
**Wine**			0.029
> once/day	3 (7.69%)	2 (16.7%)	
1–2 times/week	4 (10.3%)	1 (8.33%)	
1–3 times/week	7 (17.9%)	0 (0.00%)	
3 or more times/week	2 (5.13%)	2 (16.7%)	
Daily	22 (56.4%)	4 (33.3%)	

Continued on next page

	UT (N = 39)	AD (N = 15)	*p* overall
Never	1 (2.56%)	3 (25.0%)	
**Liquors**			0.753
1–2 times/week	4 (10.3%)	1 (8.33%)	
1–3 times/week	7 (17.9%)	4 (33.3%)	
Daily	3 (7.69%)	0 (0.00%)	
Never	25 (64.1%)	7 (58.3%)	
**Bread**			0.382
> once/day	27 (69.2%)	6 (50.0%)	
1–2 times/week	2 (5.13%)	1 (8.33%)	
3 or more times/week	2 (5.13%)	0 (0.00%)	
Daily	7 (17.9%)	5 (41.7%)	
Never	1 (2.56%)	0 (0.00%)	
**Cereals**			0.431
1–2 times/week	20 (51.3%)	9 (75.0%)	
1–3 times/week	12 (30.8%)	2 (16.7%)	
3 or more times/week	7 (17.9%)	1 (8.3 3%)	
**Cheese**			0.455
1–2 times/week	16 (41.0%)	3 (25.0%)	
1–3 times/week	10 (25.6%)	2 (16.7%)	
3 or more times/week	7 (17.9%)	3 (25.0%)	
Daily	3 (7.69%)	3 (25.0%)	
Never	3 (7.69%)	1 (8.33%)	
**Dairy**			0.907
> once/day	3 (25%)	5 (12.8%)	
1–2 times/week	1 (8.33%)	4 (10.3%)	
1–3 times/week	0	1 (2.56%)	
3 or more times/week	1 (8.33%)	5 (12.8%)	
Daily	7 (58.3%)	21 (53.8%)	
Never	0	3 (7.69%)	
**Non-Alcoholic Beverages**			0.646
> once/day	1 (8.33%)	0 (0.00%)	
1–2 times/week	1 (8.33%)	3 (7.69%)	
1–3 times/week	3 (25%)	8 (20.5%)	
3 or more times/week	0	2 (5.13%)	
Daily	2 (16.7%)	6 (15.4%)	
Never	5 (41.7%)	20 (51.3%)	
**Nuts**			1
1–2 times/week	2 (16.7%)	9 (23.1%)	
1–3 times/week	5 (41.7%)	16 (41%)	
3 or more times/week	1 (8.33%)	2 (5.13%)	
Daily	1 (8.33%)	2 (5.13%)	
Never	3 (25%)	10 (25.6%)	
**Pastries**			0.603
1–2 times/week	3 (25%)	7 (17.9%)	

Continued on next page

	UT (N = 39)	AD (N = 15)	*p* overall
1–3 times/week	5 (41.7%)	16 (41%)	
3 or more times/week	2 (16.7%)	2 (5.13%)	
Daily	1 (8.33%)	5 (12.8%)	
Never	1 (8.33%)	9 (23.1%)	
**Potatoes**			0.623
1–2 times/week	3 (25%)	17 (43.6%)	
1–3 times/week	0	1 (2.56%)	
3 or more times/week	6 (50%)	15 (38.5%)	
Daily	3 (25%)	6 (15.4%)	
**Fast Food**			1
1–3 times/week	1 (8.33%)	3 (7.69%)	
3 or more times/week	0	1 (2.56%)	
Never	11 (91.7%)	35 (89.7%)	
**Jam**			0.107
1–2 times/week	0	6 (15.4%)	
1–3 times/week	2 (16.7%)	1 (2.56%)	
3 or more times/week	1 (8.33%)	1 (2.56%)	
Daily	1 (8.33%)	1 (2.56%)	
Never	8 (66.7%)	30 (76.9%)	
**Butter**			0.867
1–2 times/week	0	1 (2.56%)	
1–3 times/week	2 (16.7%)	3 (7.69%)	
Daily	1 (8.33%)	4 (10.3%)	
Never	9 (75%)	31 (79.5%)	
**Vegetables**			0.36
> once/day	0	2 (5.13%)	
1–2 times/week	2 (16.7%)	7 (17.9%)	
3 or more times/week	3 (25%)	17 (43.6%)	
Daily	6 (50%)	13 (33.3%)	
Never	1 (8.33%)	0	
**Fruits**			0.792
> once/day	4 (33.3%)	7 (17.9%)	
1–2 times/week	2 (16.7%)	5 (12.8%)	
1–3 times/week	0	1 (2.56%)	
3 or more times/week	2 (16.7%)	9 (23.1%)	
Daily	4 (33.3%)	17 (43.6%)	
**Eggs**			0.901
1–2 times/week	9 (75%)	24 (61.5%)	
1–3 times/week	1 (8.33%)	7 (17.9%)	
3 or more times/week	2 (16.7%)	5 (12.8%)	
Daily	0	2 (5.13%)	
Never	0	1 (2.56%)	
**White Meat**			0.875
1–2 times/week	9 (75%)	26 (66.7%)	

Continued on next page

	UT (N = 39)	AD (N = 15)	*p* overall
1–3 times/week	1 (8.33%)	7 (17.9%)	
3 or more times/week	1 (8.33%)	2 (5.13%)	
Never	1 (8.33%)	4 (10.3%)	
**Red Meat**			0.303
1–2 times/week	6 (50%)	26 (66.7%)	
1–3 times/week	4 (33.3%)	10 (25.6%)	
3 or more times/week	1 (8.33%)	3 (7.69%)	
Never	1 (8.33%)	0	
**Processed Meats**			0.636
1–2 times/week	2 (16.7%)	9 (23.1%)	
1–3 times/week	6 (50%)	15 (38.5%)	
3 or more times/week	0	5 (12.8%)	
Daily	1 (8.33%)	1 (2.56%)	
Never	3 (25%)	9 (23.1%)	
**White Fish**			0.176
1–2 times/week	7 (58.3%)	26 (66.7%)	
1–3 times/week	1 (8.33%)	4 (10.3%)	
3 or more times/week	2 (16.7%)	9 (23.1%)	
Daily	1 (8.33%)	0	
Never	1 (8.33%)	0	
**Fatty Fish**			0.532
1–2 times/week	7 (58.3%)	24 (61.5%)	
1–3 times/week	3 (25%)	11 (28.2%)	
3 or more times/week	1 (8.33%)	3 (7.69%)	
Daily	1 (8.33%)	0	
Never	0	1 (2.56%)	
**Canned Food**			1
1–2 times/week	4 (33.3%)	14 (35.9%)	
1–3 times/week	6 (50%)	16 (41%)	
3 or more times/week	0	1 (2.56%)	
Never	2 (16.7%)	8 (20.5%)	

Abbreviations: UT, untreated; AD, advanced disease.

Table S2. Summary of differential abundance (species level) based on the LinDA linear model comparing advanced disease *vs*. untreated patients (reference). Reported metrics include coefficient (effect size), standard error (SE), adjusted *p*-value (FDR-corrected), mean abundance, and prevalence across samples. The table includes taxa exhibiting significant associations (adjusted $p<0.05$). Results are presented in order of decreasing statistical significance.

Variable	Coefficient	SE	Adjusted *p*-Value	Mean Abundance	Prevalence
Candidatus Alistipes merdavium	6.3	1.18	0.0002	16047.69	0.24
Unclassified Intestinimonas	−3.0	0.54	0.0002	1847.31	1
Unclassified Butyricimonas	−4.6	0.87	0.0002	3700.09	0.93
Unclassified Sutterella	−5.4	1.06	0.0002	18378.28	0.94
Sutterella wadsworthensis	−5.9	1.29	0.0009	19962.74	0.93
Unclassified Mitsuokella	5.2	1.17	0.0011	1817.98	0.5
Flavonifractor plautii	−2.2	0.49	0.0011	1328.06	1
Unclassified Lactobacillus	4.6	1.13	0.0032	5483.11	0.70

Continued on next page

Variable	Coefficient	SE	Adjusted p-Value	Mean Abundance	Prevalence
Bilophila wadsworthia	−2.6	0.65	0.0040	1281.91	0.98
Unclassified Flavonifractor	−1.8	0.46	0.0043	2011.20	1
Unclassified Bilophila	−2.0	0.54	0.0060	32455.09	1
Unclassified Enterocloster	−2.2	0.61	0.0081	1111.06	1
Alistipes indistinctus	−3.3	0.92	0.0097	960.48	0.89
Unclassified Oscillibacter	−1.4	0.39	0.0109	9820.13	1
Eggerthella sp.	2.3	0.66	0.0126	1622.57	1
Unclassified Fusobacterium	−3.8	1.11	0.01	21769.65	0.87
Podoviridae sp.	−2.0	0.59	0.0146	19212.09	1
Christensenella sp.	−3.3	1.00	0.0172	2559.26	0.70
Bacteroides eggerthii	−3.1	0.96	0.0210	3060.04	0.96
Unclassified Phascolarctobacterium	−3.4	1.08	0.0258	19355.56	1
Prevotellaceae bacterium	−3.8	1.26	0.0312	6804.31	0.80
Verrucomicrobia bacterium	−3.3	1.08	0.0312	2422.52	0.54
Clostridium perfringens	3.1	1.05	0.0362	1198.04	0.89
Unclassified Intestinibacter	3.4	1.17	0.0392	1982.41	0.93
Phocaeicola coprophilus	−3.4	1.21	0.0427	1831.70	0.76
Unclassified Odoribacter	−2.3	0.84	0.0427	41644.65	1
Unclassified Bacteroides	−1.6	0.58	0.0427	479542.24	1
Siphoviridae sp.	−0.8	0.29	0.0427	36805.54	1
Klebsiella pneumoniae	3.5	1.26	0.0445	21906.94	0.87
Streptococcus salivarius	2.6	0.95	0.0445	2637.98	1

Abbreviations: SE, standard error; FDR, false discovery rate.

Table S3. Summary of differential abundance analysis at the species level using the LinDA linear model, comparing patients in progression stage with untreated patients (reference). Metrics reported include coefficient (effect size), standard error (SE), FDR-adjusted p-value, mean abundance, and prevalence across samples. The table includes taxa exhibiting significant associations (adjusted p <0.05). Results are presented in order of decreasing statistical significance.

Variable	Coefficient	SE	Adjusted p-Value	Mean Abundance	Prevalence
Bilophila wadsworthia	−5.0	0.87	0.0001	1377.43	0.98
Unclassified Butyricimonas	−5.9	1.07	0.0001	4492.52	0.98
Unclassified Bilophila	−3.3	0.76	0.0039	35332.39	1
Unclassified Sutterella	−6.6	1.54	0.0039	23708.77	0.98
Blautia obeum	2.4	0.58	0.0046	6954.80	1
Sutterella wadsworthensis	−8.4	2.01	0.0046	26049.86	0.93
Bifidobacterium longum	5.7	1.45	0.0058	75415.32	1
Ruminococcus sp.	2.6	0.65	0.0058	22072.43	1
Alistipes indistinctus	−5.0	1.38	0.0128	1184.05	0.91
Blautia sp.	2.1	0.61	0.0161	1397.16	1
Unclassified Blautia	1.7	0.51	0.0244	38986.18	1
Anaerobutyricum hallii	2.6	0.79	0.0250	3038.45	1
Unclassified Intestinimonas	−3.0	0.91	0.0260	2427.05	1
Unclassified Mailhella	−4.7	1.56	0.0480	1608.07	0.91
Unclassified Faecalibacterium	1.5	0.50	0.0481	223790.39	1

Abbreviations: SE, standard error; FDR, false discovery rate.

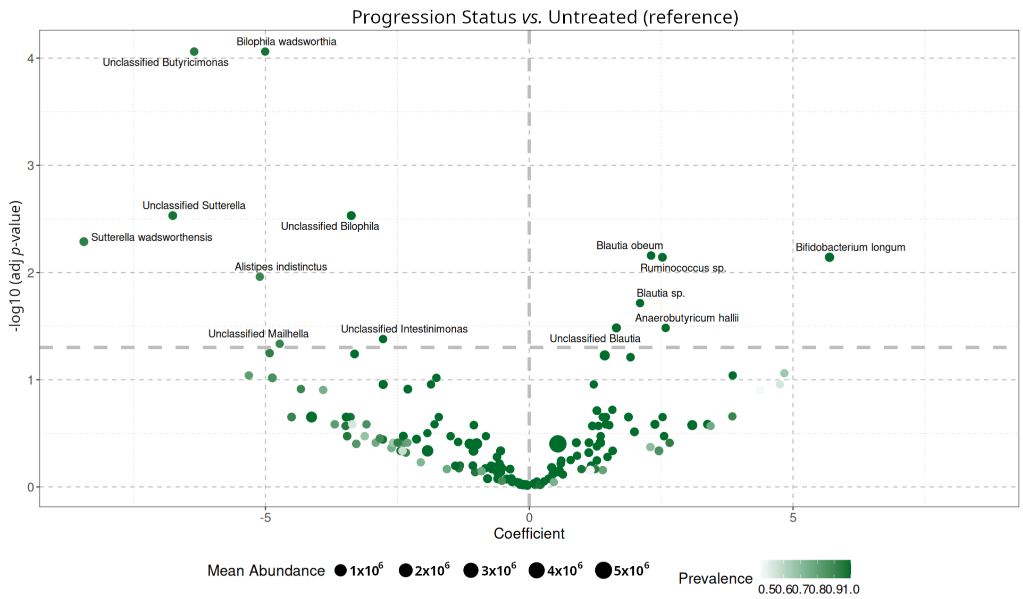

Figure S1. Gut microbiota taxa differentially represented in 5 patients with progression under ADT plus ARPI therapy compared with untreated, clinically localized PCa. Volcano plot with adjusted p-values obtained by Linear Discrimination Analysis (LinDA) in the y-axis. The x-axis shows the coefficient: enriched taxa if the coefficient is positive, depleted taxa if the coefficient is negative. The horizontal dashed line indicates the significance threshold of $p < 0.05$ after Bonferroni correction ($-\log_{10}(0.05) \approx 1.30$).

Table S4. Summary of differential abundance analysis at the species level using the LinDA linear model, comparing patients in progression stage with untreated patients (reference), adjusted by age. Metrics reported include coefficient (effect size), standard error (SE), FDR-adjusted p-value, mean abundance, and prevalence across samples. The table includes taxa exhibiting significant associations (adjusted p <0.05). Results are presented in order of decreasing statistical significance.

Variable	Coefficient	SE	Adjusted p-Value	Mean Abundance	Prevalence
Blautia obeum	3.2	0.60	0.0007	6954.80	1
Bifidobacterium longum	7.2	1.52	0.0012	75415.32	1
Bilophila wadsworthia	−4.6	0.96	0.0012	1377.43	0.98
Unclassified Butyricimonas	−5.5	1.17	0.0012	4492.52	0.98
Anaerobutyricum hallii	3.5	0.83	0.0027	3038.45	1
Blautia sp.	2.8	0.65	0.0027	1397.16	1
Unclassified Blautia	2.3	0.55	0.0027	38986.18	1
Unclassified Sutterella	−6.5	1.68	0.0079	23708.77	0.98
Ruminococcus sp.	2.5	0.70	0.0148	22072.43	1
Subdoligranulum variabile	2.7	0.76	0.0148	1399.59	1
Sutterella wadsworthensis	−7.6	2.19	0.0185	26049.86	0.93
Unclassified Faecalibacterium	1.8	0.54	0.0288	223790.39	1
Unclassified Bilophila	−2.6	0.81	0.0315	35332.39	1
Unclassified Intestinimonas	−3.0	0.99	0.0472	2427.05	1
Bacteriophage sp.	2.7	0.90	0.0513	5442.75	1
Faecalibacterium sp.	2.1	0.73	0.0592	7330.75	1
Phascolarctobacterium succinatutens	−6.2	2.15	0.0597	1242.34	0.82
Alistipes indistinctus	−4.2	1.49	0.0667	1184.05	0.91
Clostridioides difficile	1.5	0.55	0.0679	1125.36	1
Blautia wexlerae	2.1	0.81	0.0968	1632.77	1
Faecalibacterium prausnitzii	1.7	0.66	0.0968	21655.07	1
Unclassified Anaerobutyricum	2.0	0.75	0.0968	16116.34	1
Unclassified Intestinibacter	5.1	2.01	0.0968	1056.84	0.93
Unclassified Mailhella	−4.3	1.71	0.0968	1608.07	0.91
Unclassified Subdoligranulum	2.4	0.94	0.0968	4137.91	1
Verrucomicrobia bacterium	−4.8	1.89	0.0968	3208.66	0.57
Unclassified Holdemanella	3.5	1.41	0.1027	1434.14	1
CrAss-like virus sp.	−4.5	1.86	0.1049	4802.93	0.93
Subdoligranulum sp.	2.1	0.87	0.1049	1809.86	1

Continued on next page

Variable	Coefficient	SE	Adjusted *p*-Value	Mean Abundance	Prevalence
Lentisphaeria bacterium	−4.6	1.94	0.1131	2243.68	0.91
Unclassified Flavonifractor	−1.8	0.73	0.1131	2339.20	1
Unclassified Limosilactobacillus	5.0	2.10	0.1131	1596.27	0.55
Unclassified Bifidobacterium	3.1	1.31	0.1146	22154.27	1
Dorea longicatena	1.8	0.76	0.1199	2434.82	1
Unclassified Lactobacillus	4.5	1.95	0.1244	1512.05	0.64
Bacteroides thetaiotaomicron	−2.9	1.25	0.1250	18624.52	1
Flavonifractor plautii	−1.8	0.81	0.1272	1636.30	1
Streptococcus salivarius	3.6	1.60	0.1272	2519.82	1
Coprococcus comes	2.3	1.03	0.1287	24748.77	1
Phocaeicola plebeius	4.3	2.01	0.1545	30149	1
Acetobacter sp.	−5.1	2.49	0.1700	17729.93	0.91
Bacteroides salyersiae	−3.7	1.80	0.1700	6293.52	0.98
Brachyspira sp.	4.2	2.06	0.1700	1600.66	0.48
Christensenella sp.	−3.8	1.82	0.1700	3379	0.75
Unclassified Phascolarctobacterium	−3.9	1.91	0.1700	23361.84	1
Unclassified	0.9	0.46	0.1700	5291855.91	1

Abbreviations: SE, standard error; FDR, false discovery rate.

Table S5. Summary of differential abundance analysis at the species level using the LinDA linear model, comparing patients in progression stage with untreated patients (reference), adjusted by radiotherapy. Metrics reported include coefficient (effect size), standard error (SE), FDR-adjusted *p*-value, mean abundance, and prevalence across samples. The table includes taxa exhibiting significant associations (adjusted $p < 0.05$). Results are presented in order of decreasing statistical significance.

Variable	Coefficient	SE	Adjusted *p*-Value	Mean Abundance	Prevalence
Bilophila wadsworthia	−5.7	1.33	0.0175	1377.43	0.98
Bifidobacterium longum	6.4	2.24	0.2258	75415.32	1
Brachyspira sp.	8.4	2.81	0.2258	1600.66	0.48
Ruminococcus sp.	3.0	1.01	0.2258	22072.43	1
Unclassified Butyricimonas	−5.0	1.64	0.2258	4492.52	0.98
Blautia obeum	2.3	0.88	0.3176	6954.80	1
Sutterella wadsworthensis	−8.1	3.11	0.3176	26049.86	0.93
Anaerobutyricum hallii	3.0	1.22	0.3449	3038.45	1
Unclassified Bilophila	−2.8	1.17	0.3449	35332.39	1
Unclassified Intestinimonas	−3.4	1.40	0.3449	2427.05	1
Dorea longicatena	2.5	1.07	0.3796	2434.82	1
Bacteriophage sp.	2.9	1.33	0.3926	5442.75	1
Clostridia bacterium	−2.9	1.33	0.3926	2612.55	1
Unclassified Faecalibacterium	1.7	0.77	0.3926	223790.39	1
Unclassified Limosilactobacillus	6.6	2.95	0.39256	1596.27	0.55
Unclassified Blautia	1.7	0.79	0.4200	38986.18	1
Bacteroides thetaiotaomicron	−3.6	1.76	0.4513	18624.52	1
Unclassified Dorea	2.0	1.01	0.4875	34077.98	1
Unclassified Holdemanella	3.9	2.00	0.4875	1434.14	1
Unclassified Sutterella	−4.5	2.35	0.4875	23708.77	0.98
Phocaeicola plebeius	5.3	2.83	0.5160	30149	1
CrAss-like virus sp.	−4.8	2.62	0.5339	4802.93	0.93
Faecalibacterium sp.	1.9	1.03	0.5339	7330.75	1
Alistipes indistinctus	−3.5	2.12	0.5738	1184.05	0.91
Blautia wexlerae	2.0	1.17	0.5738	1632.77	1
Unclassified Alistipes	−2.2	1.31	0.5738	314607.25	1
Unclassified Anaerobutyricum	1.8	1.08	0.5738	16116.34	1
Unclassified Lactobacillus	4.9	2.80	0.5738	1512.05	0.64
Unclassified Megasphaera	−4.0	2.39	0.5738	4468.30	0.73
Bacteroides caecigallinarum	4.7	3.09	0.5892	2722.98	0.95
Blautia sp.	1.5	0.91	0.5892	1397.16	1
Christensenella sp.	−4.0	2.57	0.5892	3379	0.75
Clostridioides difficile	1.2	0.77	0.5892	1125.36	1
Clostridium celatum	−4.7	2.94	0.5892	2135.59	0.95
Unclassified Eubacterium	1.6	1.02	0.5892	7702.91	1
Unclassified Methanobrevibacter	−4.9	3.12	0.5892	5810.86	0.95

Abbreviations: SE, standard error; FDR, false discovery rate.

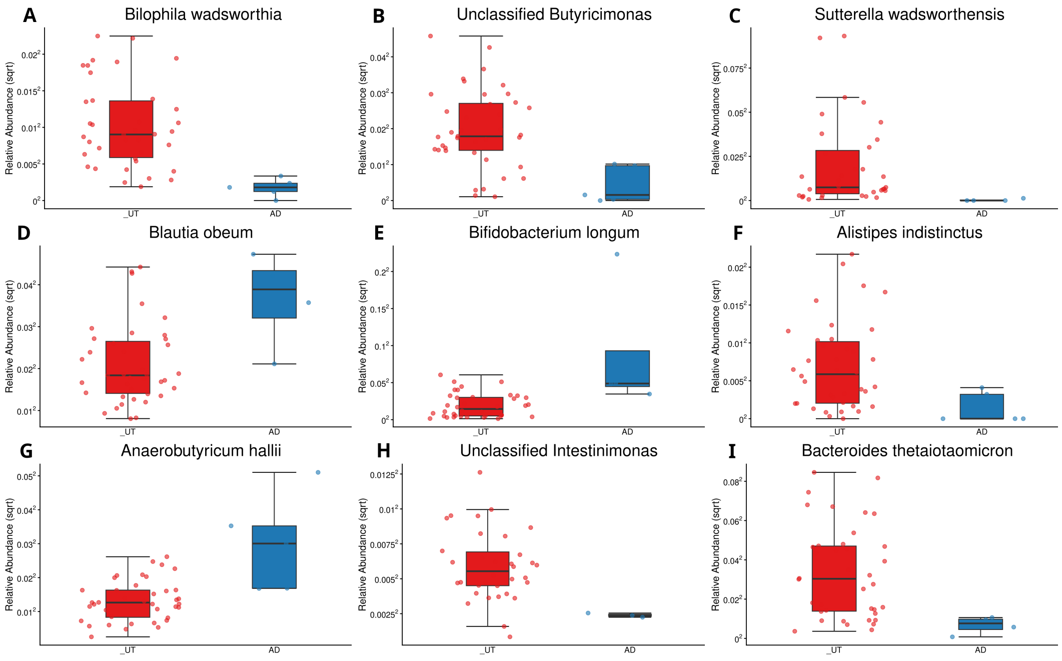

Figure S2. Differential relative abundance of bacterial taxa between patients in progression *vs*. untreated (UT) individuals. Boxplots display the distribution of square-root transformed relative abundance for taxa selected using LinDA. Each point represents an individual sample. Groups correspond to patients in progression (AD, blue) and untreated individuals (UT, red). Panels show: (A) *Bilophila wadsworthia*, (B) *unclassified Butyricimonas*, (C) *Sutterella wadsworthensis*, (D) *Blautia obeum*, (E) *Bifidobacterium longum*, (F) *Alistipes indistinctus*, (G) *Anaerobutyricum hallii*, (H) *unclassified Intestinimonas*, and (I) *Bacteroides thetaiotaomicron*. Overall, significant differences in relative abundance are observed between groups for the selected taxa.

Table S6. Significant orthologs ($p_{Adj} < 0.05$ and $Log_2FoldChange > |1|$) that coincide between the analysis performed by LinDA and DESeq2 in the AD versus UT comparative.

**KO**	**BaseMean**	$Log_2FoldChange$ **LinDA**	**lfcSE LinDA**	p_{Adj} **LinDA**	$Log_2FoldChange$ **DESeq2**	**lfcSE DESeq2**	p_{Adj} **DESeq2**	**Name**
K06617	4.07	−5.01	0.69	1.5033e-5	−3.32	0.65	1.34E-04	Rafs
K20264	5.61	−4.74	0.69	2.1138e-5	−3.64	0.58	1.28E-06	fusR
K03285	23.25	−4.44	0.90	3.6962e-3	−1.94	0.49	4.99E-03	TC.GBP
K02927	13.89	−4.20	0.81	1.8101e-3	−4.88	0.65	2.70E-10	RP-L40e
K16781	0.79	−4.17	0.80	1.8101e-3	−5.05	0.84	2.15E-06	TTC8
K17724	4.13	−4.09	0.68	2.4936e-4	−2.25	0.52	2.00E-03	IRG1
K21834	7.33	−3.91	0.70	8.1009e-4	−1.96	0.41	5.40E-04	dgcB
K14387	3.40	−3.74	0.76	3.6962e-3	−2.43	0.56	2.00E-03	SLC5A7
K11847	0.53	−3.62	0.84	1.4447e-2	−6.18	1,00	1.28E-06	USP19
K12453	1.62	−3.39	0.92	4.1746e-2	−2.36	0.77	3.98E-02	RfbS
K12900	0.37	−3.31	0.79	1.6605e-2	−5.53	1.04	4.94E-05	SRSF10
K20757	6.96	−3.29	0.58	4.9880e-4	−1.65	0.35	5.40E-04	Udp
K00696	1.07	−3.26	0.83	2.5137e-2	−2.83	0.82	1.95E-02	SPS
K09228	0.39	−3.17	0.80	2.3450e-2	−4.51	0.94	4.92E-04	KRAB
K18668	0.56	−3.17	0.71	1.2277e-2	−3.60	0.96	9.29E-03	ZC3H12
K04460	0.46	−3.12	0.83	3.5498e-2	−3.58	0.92	6.73E-03	PPP5C
K21345	2.50	−3.11	0.66	7.7256e-3	−1.43	0.42	2.12E-02	RfaE2
K20178	0.30	−3.01	0.66	1.0358e-2	−3.51	1.01	1.87E-02	VPS8
K20023	1.38	−2.72	0.60	1.0552e-2	−1.85	0.61	4.22E-02	talrD-galrD
K10219	2.27	−2.65	0.72	4.0099e-2	−1.81	0.60	4.40E-02	LigC
K21775	0.25	−2.62	0.69	3.1335e-2	−3.46	1.03	2.21E-02	LIN52
K14665	8.97	−2.54	0.44	4.9880e-4	−1.43	0.30	5.97E-04	AmxH
K16795	14.27	−2.49	0.62	2.0966e-2	−1.29	0.36	1.21E-02	PAFAH1B2-3
K22292	2.63	−2.45	0.67	4.1746e-2	−1.48	0.43	1.88E-02	MupP
K13041	16.39	−2.37	0.59	2.1135e-2	−1.34	0.35	8.02E-03	TtrR
K07101	5.73	−2.33	0.55	1.5332e-2	−1.18	0.36	2.63E-02	NA
K18901	18.21	−2.23	0.59	3.4261e-2	−1.27	0.42	4.43E-02	BpeE
K20997	4.09	−2.13	0.51	1.6605e-2	−1.36	0.36	9.86E-03	PslA
K17363	16.31	−1.79	0.49	4.0401e-2	−1.27	0.35	1.28E-02	UrdA

Continued on next page

KO	BaseMean	$Log_2FoldChange$ LinDA	lfcSE LinDA	p_{Adj} LinDA	$Log_2FoldChange$ DESeq2	lfcSE DESeq2	p_{Adj} DESeq2	Name
K07182	6.57	−1.64	0.45	4.1948e-2	−1.18	0.35	1.99E-02	CBS
K01640	4.77	−1.46	0.35	1.6605e-2	−1.37	0.35	6.09E-03	HMGCL
K17277	32.48	−1.41	0.33	1.4979e-2	−1.58	0.28	1.73E-05	EPS8
K00086	12.06	1.13	0.32	4.7715e-2	1.38	0.25	2.65E-05	DhaT
K01226	16.62	1.19	0.33	4.7715e-2	1.07	0.35	4.32E-02	TreC
K07653	1.05	1.98	0.46	1.4368e-2	1.97	0.33	2.15E-06	MprB
K12521	0.04	2.42	0.65	3.5916e-2	4.34	1.34	2.88E-02	PapF

Abbreviations: KO, Ortholog KEGG; lfcSE, $Log_2FoldChange$ Standard Error.

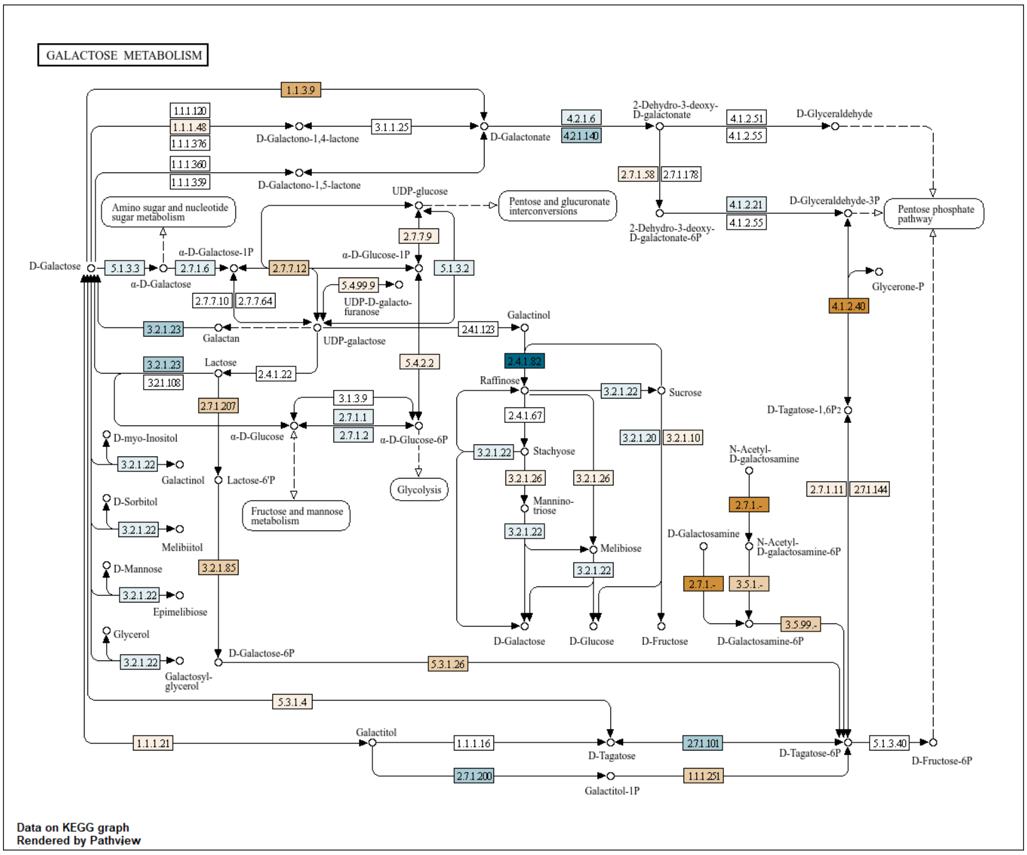

Figure S3. *Galactose metabolism pathway* (map00052). Positive $Log_2FoldChange$ values (orange) correspond to overrepresented orthologs in AD, and negative values (blue) to underrepresented orthologs in AD. Enzyme significantly underrepresented in AD patients (2.4.1.82) is shown in blue (p_{Adj} = 1.5033 × 10^{-5}).

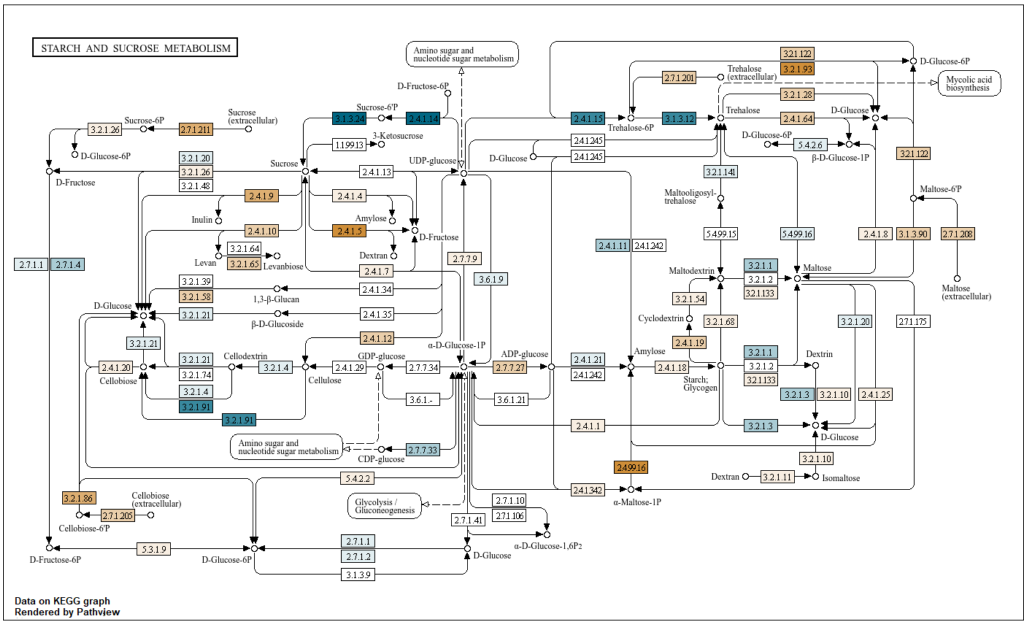

Figure S4. *Starch and sucrose metabolism pathway* (map00500). Positive $Log_2FoldChange$ values (orange) correspond to overrepresented orthologs in AD, and negative values (blue) to underrepresented orthologs in AD. Enzyme significantly underrepresented in AD patients (2.4.1.14) is shown in blue (p_{Adj} = 2.5137 × 10^{-2}).

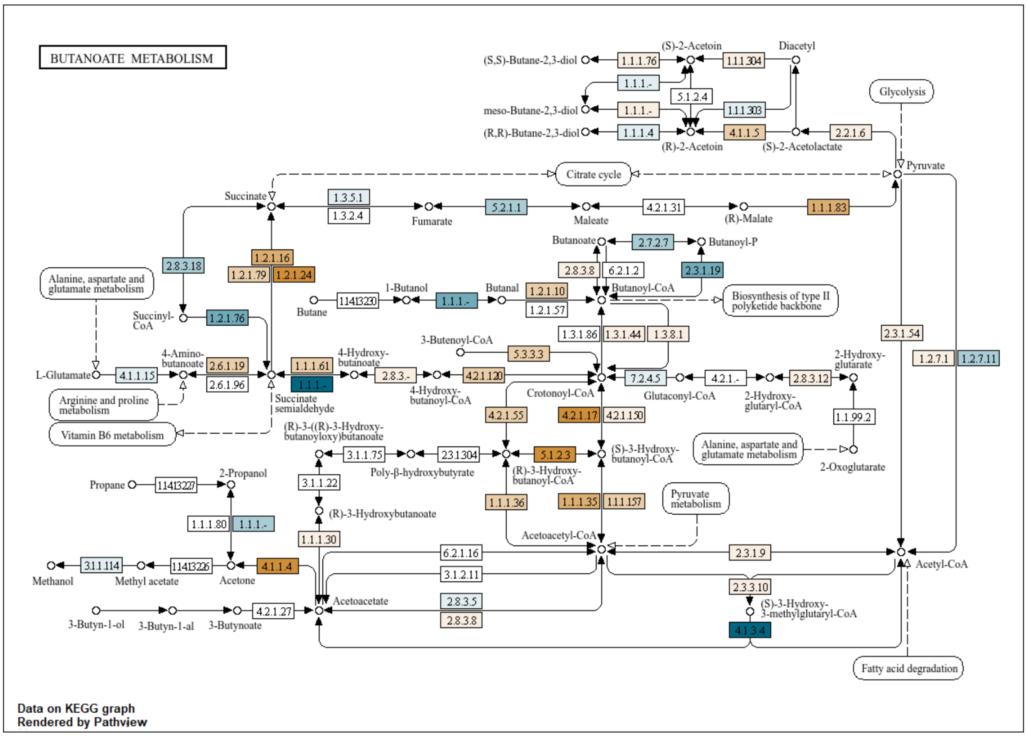

Figure S5. *Butanoate metabolism pathway* (map00650). Positive $Log_2FoldChange$ values (orange) correspond to overrepresented orthologs in AD, and negative values (blue) to underrepresented orthologs in AD. Enzymes significantly underrepresented in AD patients (4.1.3.4 and 1.1.1.-) are shown in blue (p_{Adj} = 1.6605 × 10^{-2} and p_{Adj} = 4.0099 × 10^{-2}, respectively).

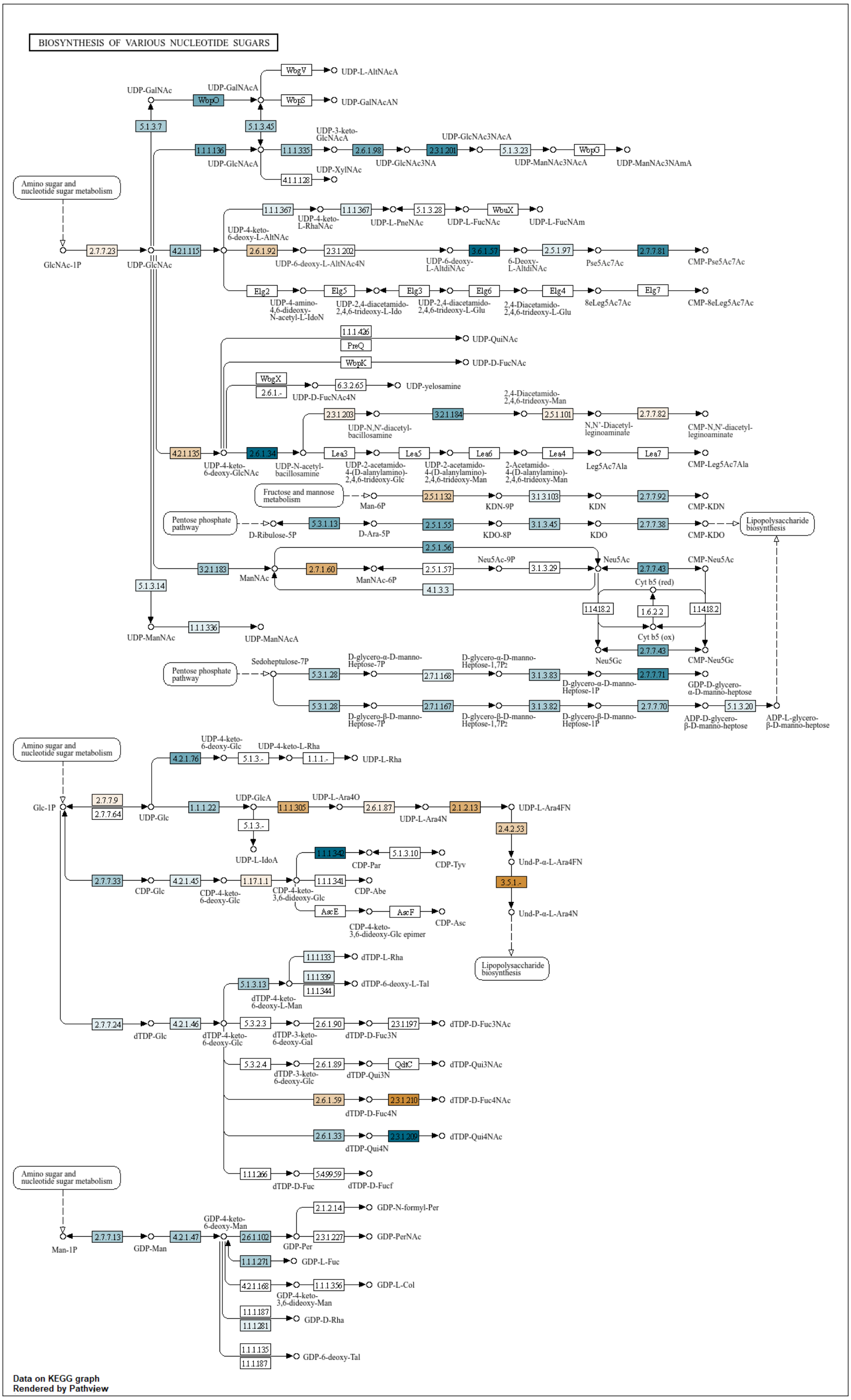

Figure S6. *Biosynthesis of various nucleotide sugars pathway* (map00541). Positive $Log_2 FoldChange$ values (orange) correspond to overrepresented orthologs in AD, and negative values (blue) to underrepresented orthologs in AD. Enzymes significantly underrepresented in AD patients (2.7.7.70 and 1.1.1.342) are shown in blue (p_{Adj} = 7.7256 × 10^{-3} and p_{Adj} = 4.1746 × 10^{-2}, respectively).

Table S7. Significant orthologs (p_{Adj} < 0.05 and $Log_2FoldChange$ > ∣1∣) that coincide between the analysis performed by LinDA and DESeq2 in the PR versus UT comparative.

KO	BaseMean	$Log_2FoldChange$ LinDA	lfcSE LinDA	p_{Adj} LinDA	$Log_2FoldChange$ DESeq2	lfcSE DESeq2	p_{Adj} DESeq2	Name
K11912	78.37	−2.64	0.59	4.18E-02	−2.38	0.57	4.46E-03	PpkA
K13598	49.94	−2.73	0.61	4.18E-02	−1.17	0.40	3.41E-02	NtrY
K02350	10.83	−5.99	1.10	1.10E-02	−4.43	0.89	7.78E-04	REV3L

Abbreviations: KO, Ortholog KEGG; lfcSE, $Log_2FoldChange$ Standard Error.

Table S8. Summary of sample metadata including study group (advanced disease [AD] or untreated [UT]), progression status (Yes/No), and corresponding European Nucleotide Archive (ENA) accession IDs for each sample.

Group	Progression	ENA ID
AD	No	ERR15104704
AD	No	ERR15104705
AD	Yes	ERR15104703
AD	Yes	ERR15104702
AD	Yes	ERR15104701
AD	Yes	ERR15104699
UT	No	ERR15104683
UT	No	ERR15104693
UT	No	ERR15104679
UT	No	ERR15104682
UT	No	ERR15104692
UT	No	ERR15104697
UT	No	ERR15104678
UT	No	ERR15104688
UT	No	ERR15104686
UT	No	ERR15104685
UT	No	ERR15104696
UT	No	ERR15104694
UT	No	ERR15104695
UT	No	ERR15104670
UT	No	ERR15104691
UT	No	ERR15104681
UT	No	ERR15104689
UT	No	ERR15104680
UT	No	ERR15104687
UT	No	ERR15104677
UT	No	ERR15104684
UT	No	ERR15104690
UT	No	ERR15104673
UT	No	ERR15104672
UT	No	ERR15104674
UT	No	ERR15104675
UT	No	ERR15104668
UT	No	ERR15104669
UT	No	ERR15104667
UT	No	ERR15104666
UT	No	ERR15104665
UT	No	ERR15104664
UT	No	ERR15104663
UT	No	ERR15104662
UT	No	ERR15104661
UT	No	ERR15104660
UT	No	ERR15104659
UT	No	ERR15104658
UT	No	ERR15104657
AD	No	ERR15104656
AD	No	ERR15104650
AD	Yes	ERR15104653
AD	No	ERR15104652
AD	No	ERR15104649
AD	No	ERR15104671
AD	No	ERR15104655
AD	No	ERR15104654
AD	No	ERR15104651
